# Synthesis and anion binding properties of phthalimide-containing corona[6]arenes

**DOI:** 10.3762/bjoc.15.193

**Published:** 2019-08-21

**Authors:** Meng-Di Gu, Yao Lu, Mei-Xiang Wang

**Affiliations:** 1 MOE Key Laboratory of Bioorganic Phosphorous Chemistry and Chemical Biology, Department of Chemistry, Tsinghua University, Beijing 100084, China

**Keywords:** anion–π interactions, coronarenes, host–guest complexation, *N*-functionalized phthalimides, O_6_-corona[3]arene[3]tetrazines

## Abstract

Functionalized O_6_-corona[3]arene[3]tetrazines were synthesized efficiently and conveniently by means of a macrocyclic condensation reaction between *N*-functionalized 3,6-dihydroxyphthalimides and 3,6-dichlorotetrazine under mild conditions in a one-pot reaction manner. The novel macrocycles exist as a mixture of rapidly interconvertible conformers in solution while in the solid state they adopt the conformation in which three phthalimide units are *cis,trans*-orientated. Acting as electron-deficient macrocyclic hosts, the synthesized O_6_-corona[3]arene[3]tetrazines self-regulated conformational structures to complex anions in the gas phase and in the solid state owing to the anion–π noncovalent interactions between anions and the tetrazine rings.

## Introduction

Synthetic macrocycles [1–2] are always attractive and important because they are unique molecular systems to study molecular recognition and the nature of noncovalent interactions. Functional macrocycles also provide essential components for the fabrication or assembly of sophisticated (supra)molecular structures [1–4], advanced materials [1–2,5–10] and machinery systems [1–2,11–12]. Moreover, the designed macrocycles are useful molecular tools in the investigation of supramolecular catalysis and reaction mechanisms [1–2,13–16].

Heteracalixaromatics or heteroatom-bridged calix(het)arenes [17–21] are synthetic macrocycles composed of heteroatoms and *meta*-(het)arenes in an alternative manner. Because of the interplay between heteroatoms and aromatic rings, heteracalixaromatics possess versatile molecular recognition properties and have found wide supramolecular applications. Very recently, we have devised coronarenes [22] simply through editing or varying the *meta*-substitution of arylenes within heteracalixaromatics into the *para*-substitution. In contrast to heteracalixaromatics which adopt generally 1,3-alternate conformations giving V-shaped cleft structures [17–21], the resulting coronarenes form, however, cylindroid cavities [22]. We have also shown that the combination of and the interplay between heteroatoms and *para*-(het)arylenes produce diverse macrocycles with coarse- and fine-tunable cavity shapes and sizes. The resulting coronarenes exhibit interesting molecular recognition properties towards anions, cations and electron-neutral organic guests [22–31].

In our previous study we have developed an efficient protocol to synthesize oxygen and sulfur-linked corona[*m*]arene[*n*]tetrazines from aromatic diol and dithiol derivatives and 3,6-dichlorotetrazine, respectively, in a simply operational one-pot reaction fashion on the basis of a nucleophilic aromatic substitution reaction [23–31]. To prepare functionalized corona[6]arenes using diethyl terephthalate as a starting material, we observed, however, the formation of a mixture of macrocyclic isomers because of a high energy barrier for the rotation of diethyl terephthalate units through the corona[6]arene macrocyclic annulus [23–24]. To circumvent the formation of structural isomers arising from the restricted rotation of aromatic fragments though the macrocycle annulus, we selected in the current study 3,6-dihydroxyphthalimide derivatives as aromatic diols to construct functionalized O_6_-corona[3]arene[3]tetrazins. Being different from terephthalate in terms of substitution pattern, we envisioned that the phthalimide unit would flip freely owing to the less steric hindrance. In addition, *N*-substituted 3,6-dihydroxyphthalimide derivatives are accessible conveniently from the reaction between various commercially available functional primary amines and 3,6-dihydoxyphthalic anhydride. The ready availability of *N*-functionalized 3,6-dihydroxyphthalimides would therefore enable the construction of functionalized coronarene macrocycles. Moreover, the electronic feature of the phthalimide would render the resulting O_6_-corona[3]arene[3]tetrazines the electron-deficient hosts to form anion-π complexes [32–47]. We report herein the synthesis, structure and anion recognition of phthalimide-containing corona[3]arene[3]tetrazines.

## Results and Discussion

**Scheme 1:**
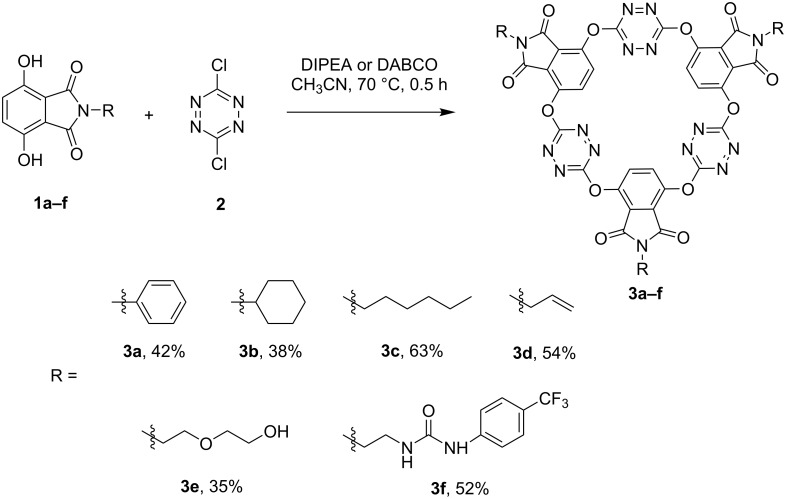
Synthesis of phthalimide-containing O_6_-corona[3]arene[3]tetrazines.

In the presence of diisopropylethylamine (DIPEA) as an acid scavenger, *N*-phenyl (**1a**), *N*-cyclohexyl (**1b**) and *N*-*n*-hexyl (**1c**)-substituted 3,6-dihydroxyphthalimides were able to react efficiently with 3,6-dichlorotetrazine [48] (**2**) under very mild conditions. The one-pot macrocyclic condensation reaction went to completion within 0.5 h to afford the corresponding O_6_-corona[3]arene[3]tetrazines **3a–c** as the only macrocyclic ring products in yields of 38% to 63%. We then prepared *N*-allyl (**1d**), *N*-(2-(2-hydroxyethoxy)ethyl (**1e**) and *N*-(2-(3-(4-(trifluoromethyl)phenyl)ureido)ethyl) (**1f**) substituted 3,6-dihydroxyphthalimides to synthesize functionalized O_6_-coronarene macrocycles. Pleasingly, the 3,6-dihydroxyphthalimide substrates **1d** and **1e** underwent the same reaction to produce corona[6]arene macrocycles **3d** and **3e** in 54% and 35% yields, respectively. Corona[3]arene[3]tetrazine bearing even a urea group (**3f**) was synthesized analogously from the reaction of **1f** with **2** when DABCO was employed as a base instead of DIPEA (Scheme 1). It was worth addressing that a chemical yield of 52% implied roughly a 90% yield for each step in these six-bond-forming synthesis. The high efficiency for the construction of a macrocyclic ring involving the formation of six new C–O bonds between two reactants was noteworthy. The successful synthesis was most probably due to both the nature of dynamic chemical bonding between tetrazine and phenolic oxygen and the high stability of corona[6]arene macrocycle under the reaction conditions.

**Figure 1:**
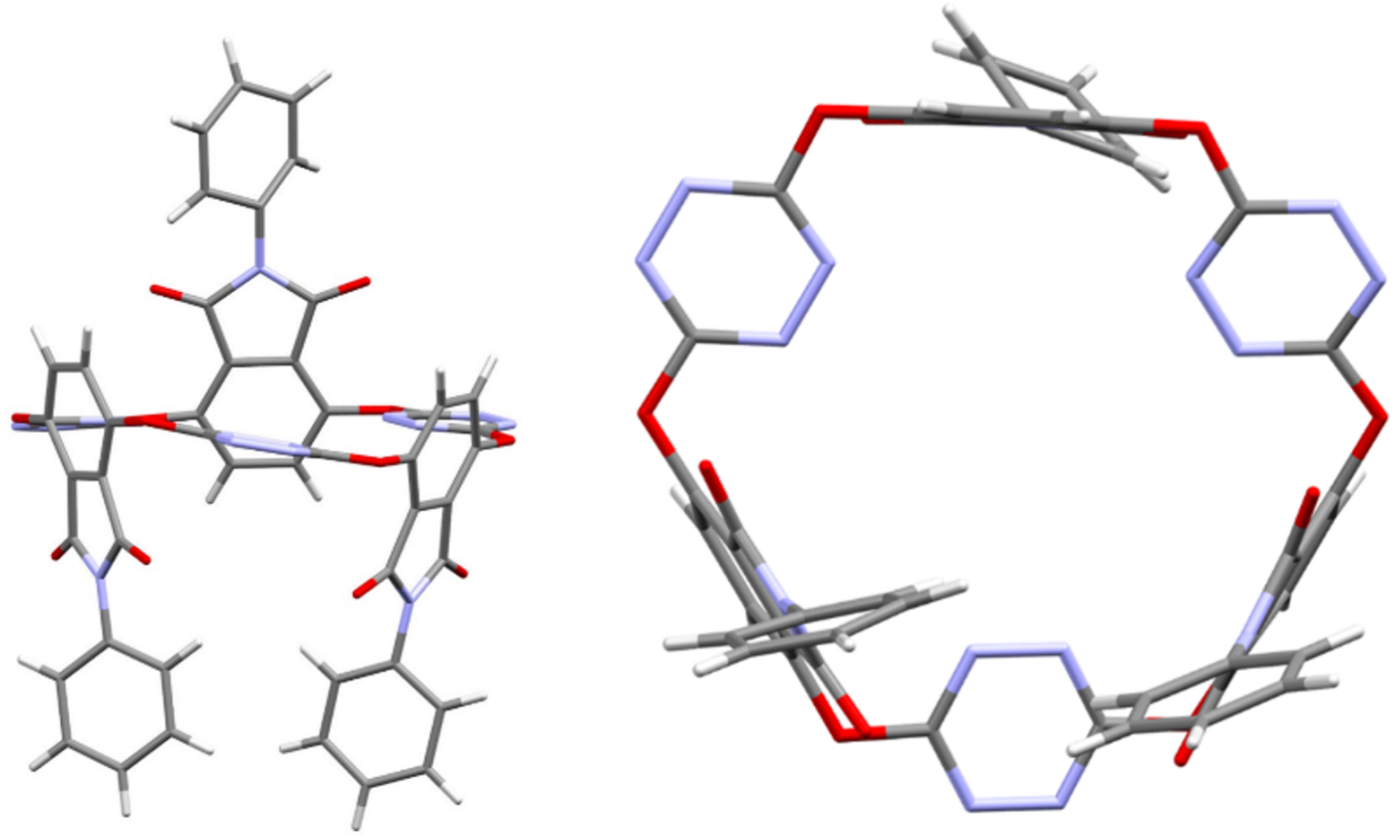
X-ray molecular structure of **3a** (CCDC 1913907) with side (left) and top (right) views. All solvent molecules are omitted for clarity.

The spectroscopic data supported the macrocyclic structure of all products. To determine the structure beyond any doubt, and also to shed light on the conformation of phthalimide-containing O_6_-corona[6]arenes in the solid state, high quality single crystals of **3a** were grown at room temperature from diffusion of diethyl ether vapor into the solution of **3a** in acetonitrile. X-ray diffraction analysis revealed that the macrocycle **3a** adopted an interesting conformation. As depicted in Figure 1, it was evident that six bridging oxygen atoms formed roughly a plane. Interestingly, three tetrazine rings are procumbent on the plane while three phthalimide units were almost perpendicular to the plane. Judging from the bond lengths, all oxygen atoms in the linking positions tended to form conjugation with their adjacent tetrazine rings rather than with the phthalimide units. Most noticeably, the three phthalimide moieties are not *cis-*configured. The orientation of one phthalimide was just opposite to that of the other two phthalimide segments.

**Figure 2:**
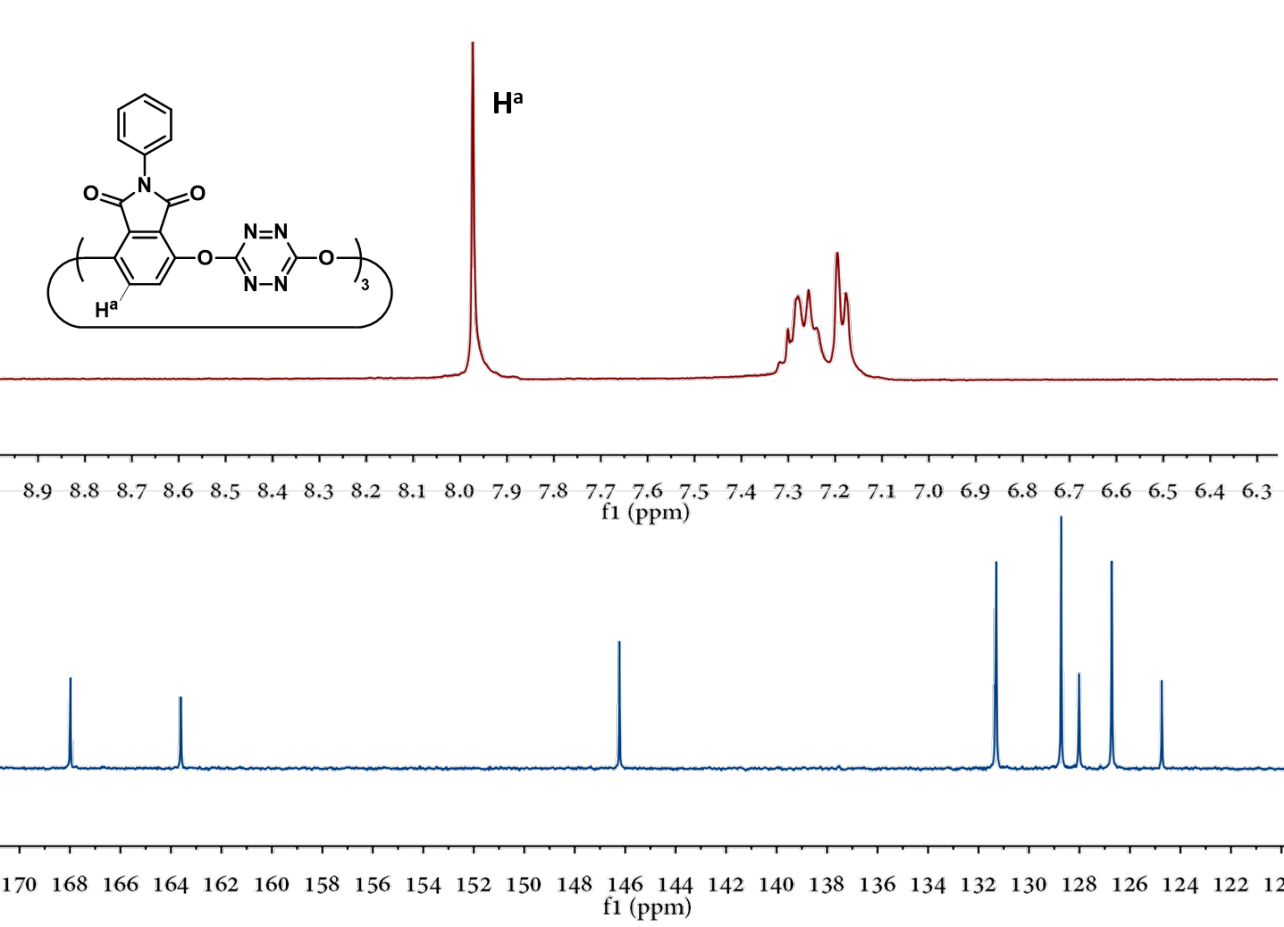
^1^H (top) and ^13^C (bottom) NMR spectra of **3a** in acetone-*d*_6_ at 25 °C.

It is important to note that corona[3]arene[3]tetrazines **3** displayed only one set of proton and carbon signals in the ^1^H and ^13^C NMR spectra, respectively, in acetone-*d*_6_ at room temperature (Figure 2). This is in sharp contradiction to O_6_-corona[3]arene[3]tetrazines composed of diethyl terephthalate which give several sets of resonance peaks [23–24]. The observation of the single set of proton and carbon resonance signals of **3** indicated the presence of the conformer with high symmetry or most likely a mixture of conformers which underwent very fast interconversion relative to the NMR time scale. In comparison to diethyl terephthalate, a phthalimide segment even containing a large substituent on the imide nitrogen atom is able to flip readily between two sides of the plane defined by bridging oxygen atoms. The conformational fluxionality of the macrocyclic ring would be beneficial to molecular recognition.

**Figure 3:**
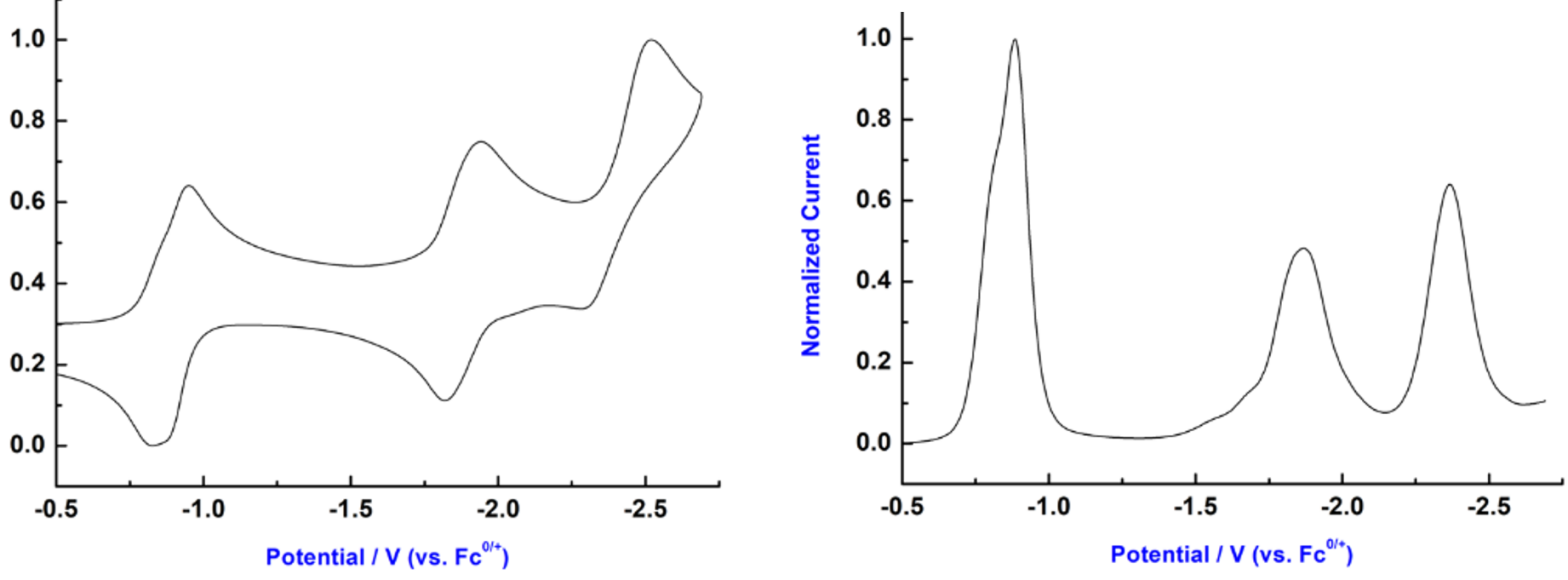
Normalized cyclic voltammograms (left) and differential pulse voltammograms (right) of **3a**. CV and DPV were recorded (scan rate 100 mV·s^−1^) using a glassy carbon electrode. All of the experiments were performed at 298 K in argon-purged MeCN solutions 0.5 mM) with 0.01 M of [Bu_4_N]^+^[PF_6_]^−^ as the supporting electrolyte. Potentials were recorded versus Fc^+^/Fc.

To gain a deep insight into the redox properties of phthalimide-bearing O_6_-corona[3]arene[3]tetrazines, a cyclic voltammogram (CV) and a differential pulse voltammogram of **3a** were recorded. As depicted in Figure 3, macrocycle **3a** undergoes a reversible sequential one-electron redox process at −811, −883, −1871, and −2367 mV. The electrochemical result indicated the occurrence of electronic communication between aromatic rings. The potential for the first redox process (−811 mV) obtained from CV and DPV revealed the electron deficiency of the macrocycle.

**Figure 4:**
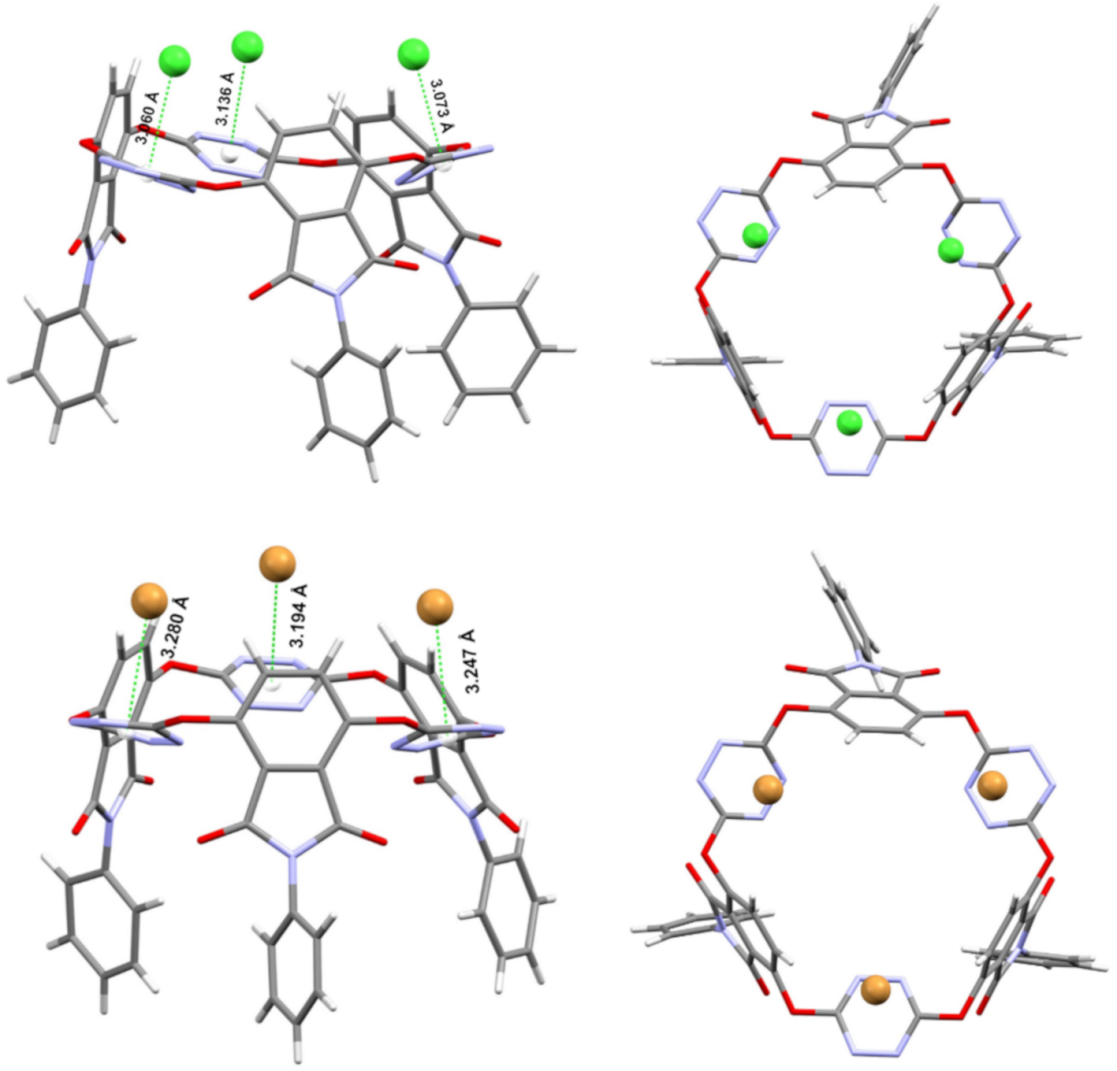
X-ray molecular structures of complexes (*n*-Bu_4_NCl)_3_-**3a** (1913908) (top) and (*n*-Bu_4_NBr)_3_-**3a** (1913909) (bottom) with side (left) and top (right) views. Cations and solvent molecules are omitted for clarity.

The resulting electron-deficient O_6_-corona[3]arene[3]tetrazines prompted us to investigate their anion binding behaviour. Taking compound **3a** as a representative, we examined the interaction of macrocycles **3** with anions of tetra-*n*-butylammonium salts by means of electron spray ionization (ESI) mass spectrometry. It was found that the mass spectra of mixed samples of **3a** with *n*-Bu_4_NX gave ion peaks corresponding to [**3a** − X]^−^, [**3a**-*n*-Bu_4_N − 2X]^−^ and [**3a**-*n*-2Bu_4_N − 3X]^−^ complexes in which anions X^−^ included spherical Cl^−^, Br^−^, I^−^, linear SCN^−^, planar triangle NO_3_^−^, tetrahedral BF_4_^−^ and octahedral PF_6_^−^ (see Supporting Information File 1). These results demonstrated clearly the outstanding ability of **3a** to bind various anion species in gas phase. To our delight, host molecule **3a** co-crystalized with *n*-Bu_4_NX (X = Cl, Br) from diffusion of diethyl ether vapor into ethyl acetate solution at ambient temperature to give single crystals of the host–guest complexes (*n*-Bu_4_NX)_3_-**3a** (X = Cl, Br). X-ray crystallography then allowed us to understand the host–guest interactions at the molecular level. As illustrated in the molecular structures in Figure 4, above the centroid of each tetrazine ring there resided a chloride or a bromide. The distance of the anion to the centroid of tetrazine ranged from 3.060 Å to 3.136 Å (Cl^−^) or from 3.194 Å to 3.280 Å (Br^−^). The location of anion above the tetrazine centroid with the shorter distance than the sum of van der Waals radius indicated explicitly the typical noncovalent anion–π attraction. It is interesting to point out that in the host–guest complexes, all phthalimide units or their *N*-phenyl substituents became parallelly aligned (Figure 4). It seems that macrocyclic host **3a** changed from its *cis,trans*-conformation (Figure 2) to the *cis,cis* one in order to best complex guest species. Another noteworthy structure feature was the deformation of all tetrazine rings. Upon complexation with a halide, the planar aromatic ring adopted a heavily pinched boat conformation, a result consistent with theoretical prediction [49]. We also examined the host–guest interaction in solution phase employing NMR and UV–vis spectroscopy and fluorescence technology. Unfortunately, titration of the host with the guest species did not cause appreciable spectral changes. Isotherm titration calorimetry (ITC) did not give satisfactory results either.

## Conclusion

In summary, we have synthesized phthalimide-containing functionalized O_6_-corona[3]arene[3]tetrazines by means of a one-pot macrocyclic condensation reaction between *N*-functionalized 3,6-dihydroxyphthalimides and 3,6-dichlorotetrazine. The unprecedented macrocycles exist as a mixture of rapidly inter-convertible conformers in solution relative to the NMR time scale. The novel O_6_-corona[3]arene[3]tetrazines self-regulated conformational structures to complex anions in the gas phase and in the solid state owing to the anion–π noncovalent interactions between anions and the tetrazine rings. The easy accessibility, cylindroid cavity and diverse functionality would engender phthalimide-containing functionalized O_6_-corona[3]arene[3]tetrazines a useful macrocyclic platform for the study of supramolecular chemistry. Applications of the phthalimide-containing functionalized O_6_-corona[3]arene[3]tetrazines are being actively perused and results will be reported in due course.

## Experimental

**General procedure for the synthesis of 3a–e.** To a solution of DIPEA (2.1 mmol) in acetonitrile (150 mL) which was pre-heated to 70 °C was added dropwise a solution of 3,6-dihydroxyphthalimide derivatives **1a–e** (1 mmol) and 3,6-dichlorotetrazine (**2**, 1 mmol) in acetonitrile (25 mL) during 25 min. After being stirred for another 5 min, the reaction was quenched by cooling down the mixture to ambient temperature and adding water (200 mL). The resulting mixture was neutralized with dilute hydrochloric acid (1 M) and extracted with EtOAc (3 × 150 mL). The combined organic solution was washed with brine (3 × 200 mL) and then dried over anhydrous Na_2_SO_4_. After filtration and concentration, the residue was chromatographed on a silica gel column using a mixture of DCM and EtOAc (v/v = 100:1) as an eluent to give product **3a–e**.

**3a:** 140 mg, yield 42%, red solid, mp 275 °C (decomp.); ^1^H NMR (400 MHz, acetone-*d*_6_, 25 °C) δ 8.00 (s, 6H), 7.20–7.34 (m, 15H); ^13^C NMR (101 MHz, acetone-*d*_6_, 25 °C) δ 168.8, 164.4, 147.1, 132.2, 132.1, 129.6, 128.8, 127.5, 125.6; IR (KBr, cm^−1^) ν: 3078, 1777, 1724, 1494, 1384, 1230, 1115, 955; HRMS-APCI: [M + H]^+^ calcd for C_48_H_22_N_15_O_12_, 1000.1567; found, 1000.1553; anal. calcd for C_48_H_21_N_15_O_12_: C, 57.66; H, 2.12; N, 21.01; found: C, 57.71; H, 2.08; N, 20.70.

**3b:** 129 mg, yield 38%, red solid, mp 275 °C (decomp.); ^1^H NMR (400 MHz, MeCN-*d*_3_, 25 °C) δ 7.72 (s, 6H), 3.77–3.70 (m, 3H), 1.83–1.76 (m,6H), 1.71–1.68 (m, 6H), 1.25–1.04 (m, 9H); ^13^C NMR (101 MHz, MeCN-*d*_3_, 25 °C) δ 168.6, 165.5, 146.4, 131.5, 125.5, 52.0, 30.0, 26.5, 25.8; IR (KBr, cm^−1^) ν: 2934, 2858, 1772, 1715, 1382, 1228, 955; HRMS-APCI: [M + H]^+^ calcd for C_48_H_40_N_15_O_12_, 1018.2975; found, 1018.2954; anal. calcd for C_48_H_39_N_15_O_12_: C, 56.64; H, 3.86; N, 20.64; found: C, 56.51; H, 3.83; N, 20.24.

**3c:** 215 mg, yield 63%, red solid, mp 275 °C (decomp.); ^1^H NMR (400 MHz, CDCl_3_, 25 °C) δ 7.63 (s, 6H), 3.38 (t, *J* = 6.8 Hz, 6H), 1.54–1.48 (m, 6H), 1.23–1.19 (m, 18H), 0.82 (t, *J* = 6.8 Hz, 9H); ^13^C NMR (101 MHz, CDCl_3_, 25 °C) δ 167.4, 164.4, 145.5, 130.1, 124.7, 38.6, 31.2, 29.7, 28.1, 26.4, 22.4, 13.9; IR (KBr, cm^−1^) ν: 3081, 2931, 2859, 1774, 1717, 1494, 1440, 1417, 1379, 1227, 1065, 954; HRMS-APCI: [M + H]^+^ calcd for C_48_H_46_N_15_O_12_, 1024.3445; found, 1024.3437; anal. calcd for C_48_H_45_N_15_O_12_·acetone: C, 56.61; H, 4.75; N, 19.42; found: C, 56.17; H, 4.66; N, 19.34.

**3d:** 160 mg, yield 54%, red solid, mp > 300 °C; ^1^H NMR (400 MHz, CDCl_3_, 25 °C) δ 7.66 (s, 6H), 5.70–5.64 (m, 3H), 5.12–5.07 (m, 6H), 4.00 (d, *J* = 6.0 Hz, 3H); ^13^C NMR (101 MHz, CDCl_3_, 25 °C) δ 167.4, 163.8, 145.6, 130.4, 130.2, 124.6, 118.6, 40.5; IR (KBr, cm^−1^) ν: 3288, 1781, 1725, 1428, 1380, 1344, 1229, 1229, 1122, 954, 924; HRMS-APCI: [M + H]^+^ calcd for C_39_H_22_N_15_O_12_, 892.1567; found, 892.1563. anal. calcd for C_39_H_21_N_15_O_12_·0.5H_2_O: C, 52.01; H, 2.46; N, 23.33; found: C, 52.39; H, 2.11; N, 22.94.

**3e:** 121 mg, yield 35%, red solid, mp 182–186 °C; ^1^H NMR (400 MHz, acetone-*d*_6_, 25 °C) δ 7.95 (s, 6H), 3.50–3.55 (m, 12H), 3.34–3.41 (m, 12H), 2.83 (s, 3H); ^13^C NMR (101 MHz, acetone-*d*_6_, 25 °C) δ 168.7, 165.3, 146.6, 131.8, 125.7, 73.2, 67.8, 61.8, 38.6; IR (KBr, cm^−1^) ν: 3472, 1776, 1716, 1382, 1228, 956; HRMS-APCI: [M + Na]^+^ calcd for C_42_H_33_N_15_O_18_, 1058.2020; found, 1058.2029; anal. calcd for C_42_H_33_N_15_O_18_·H_2_O: C, 48.87; H, 3.35; N, 19.94; found: C, 48.12; H, 3.01; N, 19.90.

**Synthesis of 3f:** To a solution of DABCO (2.1 mmol) in acetonitrile (150 mL) which was pre-heated to 70 °C was added dropwise a solution of **1f** (1 mmol) and 3,6-dichlorotetrazine (**2**, mmol) in acetonitrile (25 mL) during 25 min. After being stirred for another 5 min, the reaction was quenched by cooling the mixture down to ambient temperature and adding water (200 mL). The resulting mixture was neutralized with dilute hydrochloric acid (1 M) and extracted with EtOAc (3 × 150 mL). The combined organic solution was washed with brine (3 × 200 mL) and then dried over anhydrous Na_2_SO_4_. After filtration and concentration, the residue was chromatographed on a silica gel column using a mixture of DCM and EtOAc (v/v = 25:1) as an eluent to give product **3f** (261 mg, yield 52%) as a red solid, mp 207–210 °C; ^1^H NMR (400 MHz, DMSO-*d*_6_, 25 °C) δ 8.98 (s, 3H), 7.88 (s, 5H), 7.53 (s, 12H), 6.23 (t, *J* = 5.7 Hz, 3H), 3.36 (m, 6H), 3.01 (m, 6H), 1.60 (t, *J* = 6.4 Hz, 6H); ^19^F NMR (376 MHz, DMSO-*d*_6_, 25 °C) δ 59.8; ^13^C NMR (101 MHz, MeCN-*d*_3_, 25 °C) δ 167.1, 164.5, 154.8, 145.0, 144.2, 131.2, 125.9, 124.7 (q,^1^*J*(C, F) = 271.6 Hz), 124.5, 120.9 (q, ^2^*J*(C, F) = 31.8 Hz), 117.1, 36.5, 35.5, 28.2; IR (KBr, cm^−1^) ν: 3372, 3080, 2937, 1775, 1717, 1603, 1545, 1382, 1325, 1228, 1113, 1066. HRMS-APCI: [M − H]^−^ calcd for C_63_H_41_N_21_O_15_F_9_, 1502.2953; found, 1502.2972; anal. calcd for C_63_H_42_F_9_N_21_O_15_·H_2_O: C, 49.71; H, 2.91; N, 19.32. found: C, 49.42; H, 3.04; N, 19.01.

## Supporting Information

File 1 Experimental procedures, characterization of products and copies of mass and NMR spectra.
