# Supplementary material for: Synthesis and anion binding properties of phthalimide-containing corona[6]arenes
Source: Beilstein J Org Chem. 2019 Aug 21;15:1976–83. doi: 10.3762/bjoc.15.193 (PMC6720058; doi:10.3762/bjoc.15.193)
Supplement: File 1 — Experimental procedures, characterization of products and copies of mass and NMR spectra. [file Beilstein_J_Org_Chem-15-1976-s001.pdf]

BEILSTEIN JOURNAL OF ORGANIC CHEMISTRY

Supporting Information

for

Synthesis and anion binding properties of phthalimide-containing corona[6]arenes

Meng-Di Gu, Yao Lu and Mei-Xiang Wang

Beilstein J. Org. Chem. **2019,** *15,* 1976–1983. doi:10.3762/bjoc.15.193

Experimental procedures, characterization of products and copies of mass and NMR spectra

License and Terms: This is a supporting information file under the terms of the Creative Commons Attribution License (http://creativecommons.org/licenses/by/4.0). Please note that the reuse, redistribution and reproduction in particular requires that the authors and source are credited.
The license is subject to the *Beilstein Journal of Organic Chemistry* terms and conditions: (https://www.beilstein-journals.org/bjoc)

1. General information S2

2. Experimental procedures and characterization of products S2–S5

3. Reference S5

4. Mass spectra of 3a with *n*-Bu_4NX S6–S13

5. Copies of ^{1}H and ^{13}C NMR spectra S14–S35

1. General information

All commercially available reagents were used as received. TLC analysis was performed on pre-coated, glass-backed silica gel plates and visualized with UV light. Flash column chromatography was performed on silica gel (100–200). Anhydrous acetonitrile was dried by 4 Å molecular sieves. ^{1}H NMR and ^{13}C NMR spectra were recorded using 400 MHz spectrometers. Chemical shifts are reported in ppm versus either tetramethylsilane or the residual solvent resonance used as an internal standard. Abbreviations are used in the description of NMR data as follows: chemical shift (δ, ppm), multiplicity (s = singlet, d = doublet, t = triplet, m = multiplet), coupling constant (*J*, Hz). Infrared spectra were recorded using a FTIR spectrometer with KBr discs in the 4000–400 cm^{-}1 region. Mass and elemental analysis was performed at the Institute of Chemistry, CAS. Melting points are uncorrected.

2. Experimental procedures and characterization of products.

The synthesis **1a**, **1b** was conducted following previously reported methods[1].

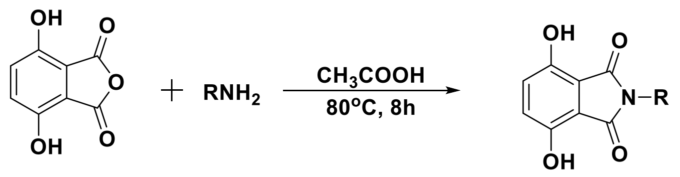

1c-e

Synthesis of ***1c–e*** *:* 3, 6-Dihydroxyphthalic anhydride (500 mg, 2.83 mmol) was dissolved in AcOH (5 mL) with corresponding amine (5.66mmol) being added and the mixture was heated at 80 °C for 8 h. The mixture was then cooled gradually to room temperature, and water (50 mL) was added. The resulting mixture was extracted with ethyl acetate (4 × 50 mL). The combined organic phase was washed with brine

(3 × 100 mL), and dried over anhydrous Na_2SO_4. After filtration and removal of the solvent, the residue was chromatographed on a silica gel column with a mixture of petroleum ether and ethyl acetate as the mobile phase to give pure product **1c–e**.

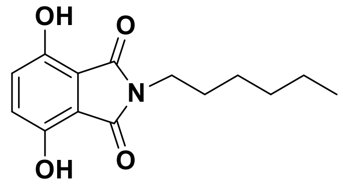

1c

1c (536 mg, yield 76%): yellow solid, mp decompose at 275°C; ^{1}H NMR (400 MHz, DMSO-*d6*, 25 °C) δ 10.10 (s, 2H), 7.05 (s, 2H), 3.45 (t, *J* = 7.1 Hz, 2H), 1.52 (t, *J* = 6.6 Hz, 2H), 1.25 (s, 6H), 0.85 (t, *J* = 6.9 Hz, 3H); ^{13}C NMR (101 MHz, DMSO-*d6*, 25 °C) δ 166.4,147.9, 125.8, 114.0, 36.6, 30.8, 27.9, 25.9, 22.0, 13.9; IR (KBr, cm^{-1}) ν 3414, 2955, 2923, 2854, 1746, 1675, 1497, 1160, 928. HRMS- APCI calcd. for $C_{14}H_{16}NO_4$ [M-H]$^-$: 262.1085; Found,262.1079.

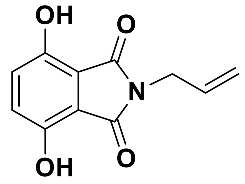

1d

1d (341 mg, yield 55%): yellow solid, mp 193-195 °C; ^{1}H NMR (400 MHz, $CDCl_3$, 25 °C) δ 7.10 (s, 2H), 7.09 (s, 2H), 5.91-5.82 (m, 1H), 5.29-5.21 (m, 2H), 4.23 (td, *J* = 3.4, 1.8 Hz, 2H); ^{13}C NMR (101 MHz, $CDCl_3$, 25 °C) δ 169.0, 148.6, 131.2, 126.1, 118.0, 112.2, 39.7; IR (KBr, cm^{-1}) ν 3222, 1739, 1681, 1641, 1491, 1434, 1382, 1339, 1291, 1169, 1064, 954, 936,913. HRMS- APCI calcd. for $C_{11}H_8$ O_4N [M-H]$^-$: -218.0459; Found, 218.0452.

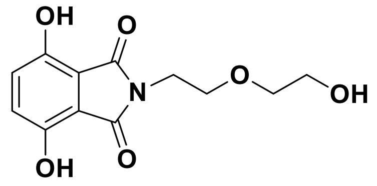

1e

1e (204 mg, yield 27%): yellow solid, mp 136-138°C; ^{1}H NMR (400 MHz, DMSO-d_6, 25 °C) δ 10.20 (s, 2H), 7.06 (s, 2H), 4.57 (d, J = 5.0 Hz, 1H), 3.53-3.64 (m, 4H), 3.40-3.44 (m, 4H); ^{13}C NMR (101 MHz, DMSO-d_6, 25 °C) δ 166.3, 148.0, 125.9, 114.1, 71.9, 67.1, 60.1, 36.4; IR (KBr, cm^{-1}) ν 2918, 1750, 1694, 1644, 1493, 1385, 1283, 1261, 1172, 1010, 933, 827, 761. HRMS- APCI calcd. for $C_{14}H_{16}NO_4$ $[M-H]^-$: 266.0670; Found, 266.0667.

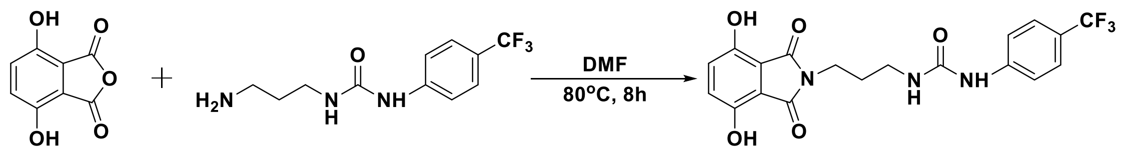

4f **1f**

Synthesis of ***1f*** *:* 3, 6-Dihydroxyphthalic anhydride (250 mg, 1.42 mmol) was dissolved in DMF (3 mL) with **4f** (563 mg, 2.13 mmol) being added and the mixture was heated at 80 °C for 8 h. The mixture was then cooled gradually to room temperature, water (50 mL) was added. The resulting mixture was extracted with ethyl acetate (4 × 50 mL). The combined organic phase was washed with brine (3 × 100 mL), and dried over anhydrous Na_2SO_4. After filtration and removal of the solvent, the residue was chromatographed on a silica gel column with a mixture of petroleum ether and ethyl acetate as the mobile phase to give pure product **1f** (162 mg, 27% yield) as yellow solid: mp 219-222 °C; ^{1}H NMR (400 MHz, DMSO-d_6, 25 °C) δ 10.15 (s, 2H), 9.03 (s, 1H),

7.54-7.60 (m, 4H), 7.06 (s, 2H), 6.34 (t, *J* = 5.7 Hz, 1H), 3.50 (t, *J* = 6.9 Hz, 2H), 3.08 (q, *J* = 6.4 Hz, 2H), 1.66-1.73 (m, 2H); ^{19}F NMR (376 MHz, DMSO-*d*$_6$, 25 ºC) δ -59.785; ^{13}C NMR (101 MHz, DMSO-*d*$_6$, 25 ºC) δ 166.6, 154.8, 148.0, 144.3, 126.0, 125.9, 122.0 (q, ^1J (C,F) = 270.6 Hz), 120.89 (q, ^2J (C,F) = 32.7Hz), 117.2, 114.1, 36.8, 34.5, 28.9; IR (KBr, cm^{-1}) ν 3314, 2926, 1671, 1640, 1333, 1161, 1132, 931. HRMS-ESI calcd. for $C_{19}H_{15}F_3N_3O_5$, $[M-H]^-$ 422.0969; Found, 422.0966.

3. Reference

1.Kenta Kanosuea, Shinji Ando, *Phys. Chem. Chem. Phys.*, **2015**, *17*, 30659 - 30669

4. Mass spectra of 3a with *n*-Bu_4NX

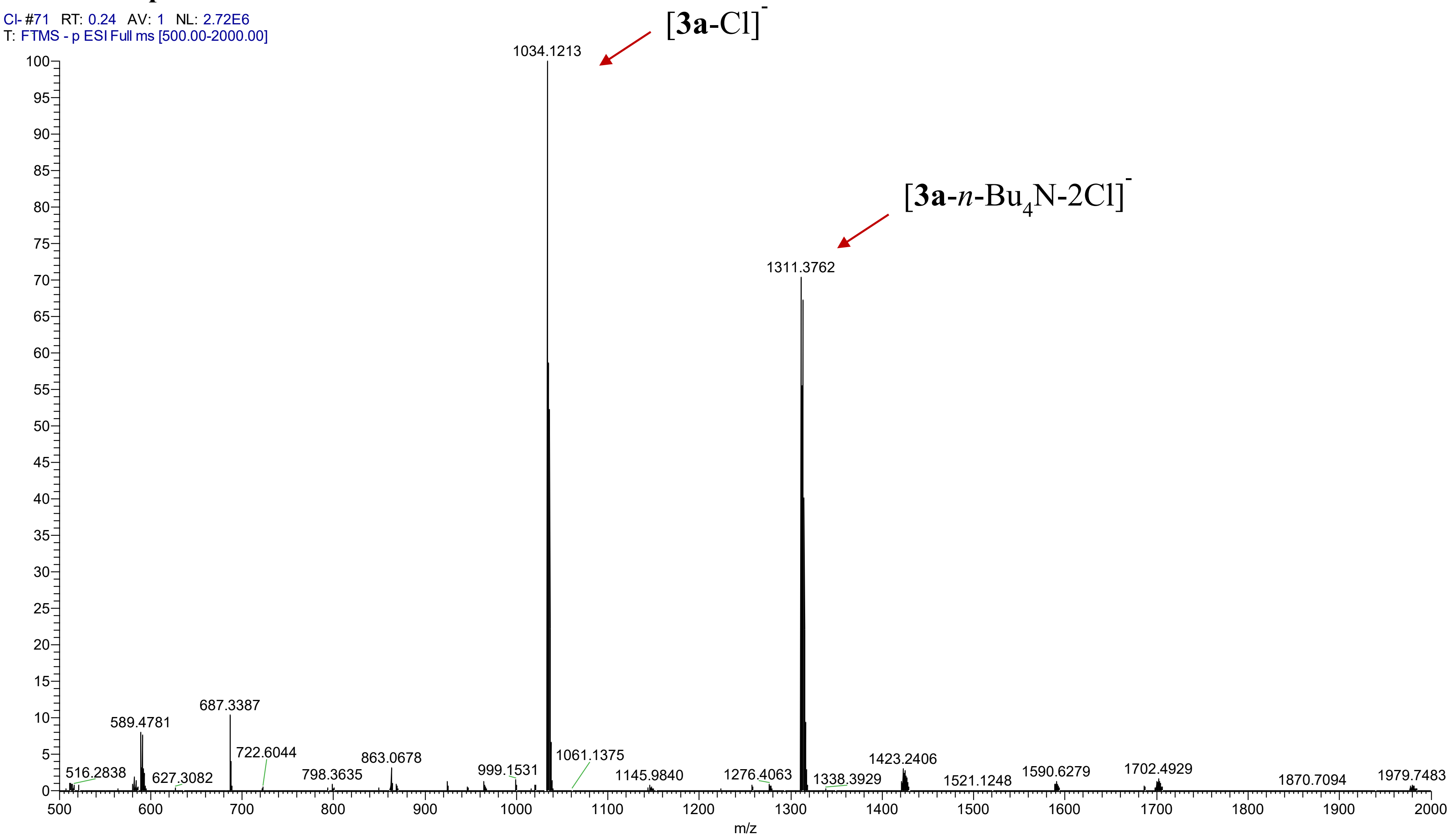

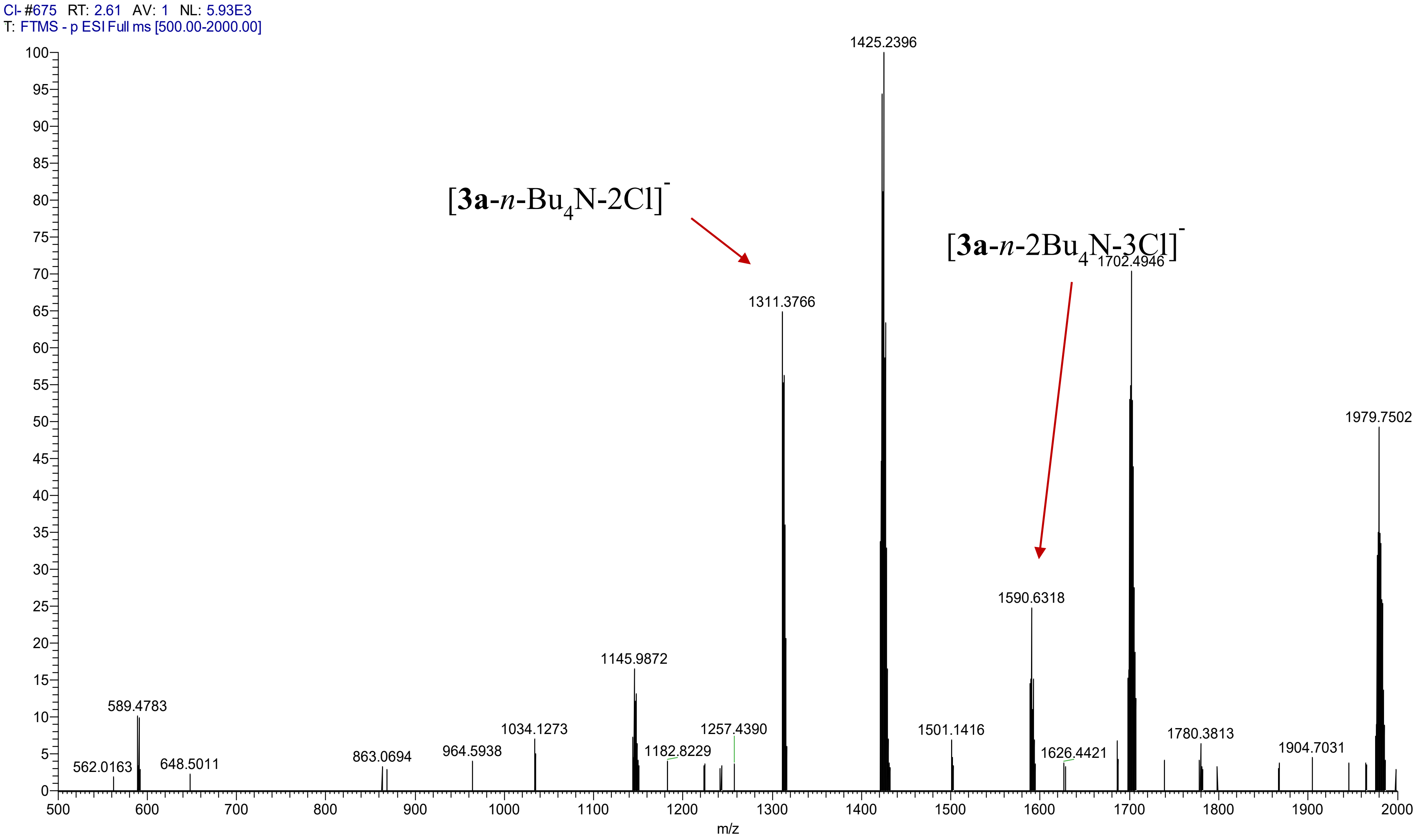
Cl- #675 RT: 2.61 AV: 1 NL: 5.93E3
T: FTMS - p ESI Full ms [500.00-2000.00]
[3a-n-Bu4N-2Cl]-
[3a-n-2Bu4N-3Cl]-
1425.2396
1311.3766
1702.4946
1979.7502
1590.6318
1145.9872
589.4783
1034.1273
1257.4390
1501.1416
1780.3813
1904.7031
1182.8229
1626.4421
964.5938
863.0694
562.0163
648.5011
m/z

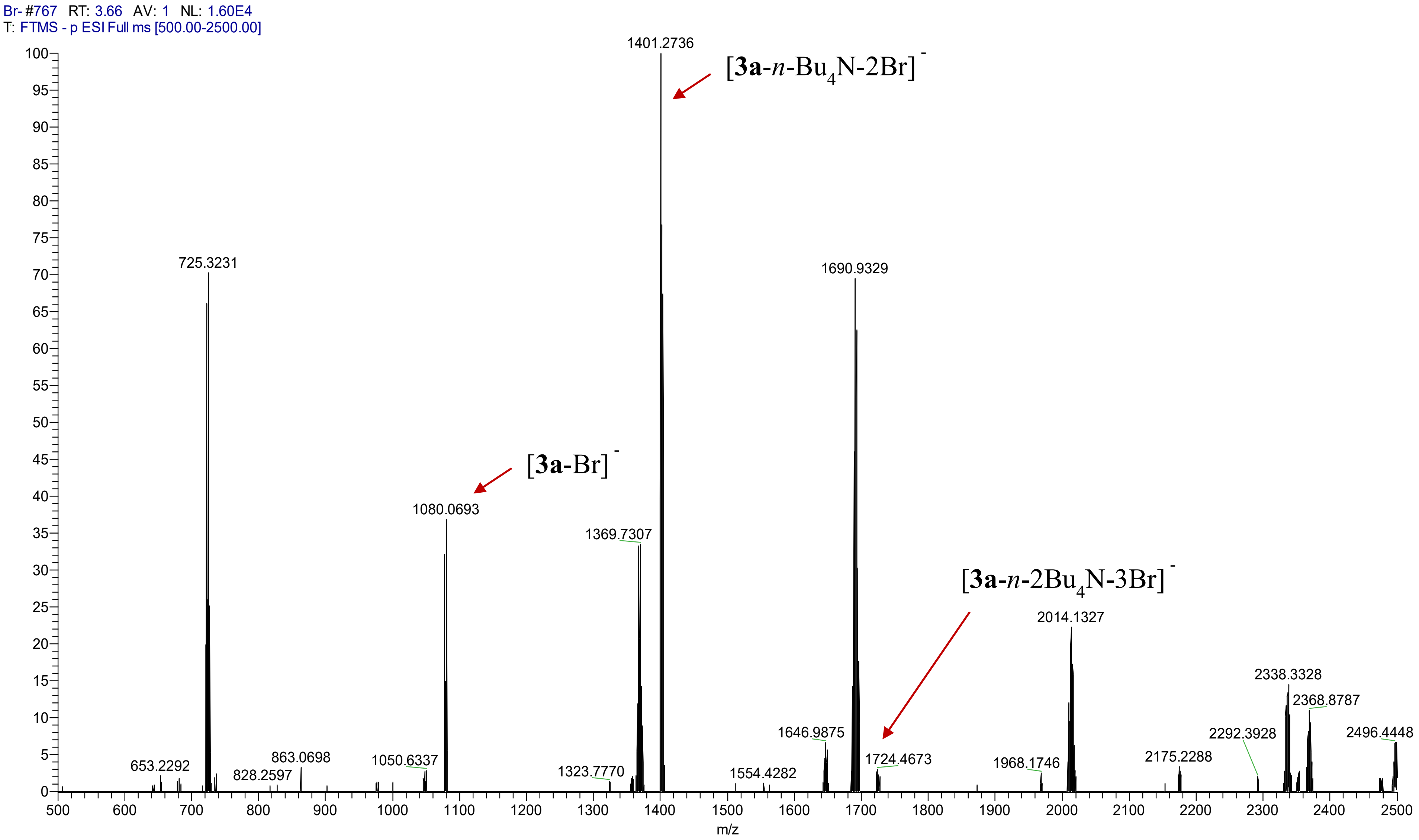
Br- #767 RT: 3.66 AV: 1 NL: 1.60E4
T: FTMS - p ESI Full ms [500.00-2500.00]
1401.2736
[3a-n-Bu4N-2Br]-
725.3231
1690.9329
[3a-Br]-
1080.0693
1369.7307
[3a-n-2Bu4N-3Br]-
2014.1327
2338.3328
2368.8787
2292.3928
2496.4448
1646.9875
1724.4673
1968.1746
2175.2288
653.2292
863.0698
828.2597
1050.6337
1323.7770
1554.4282
m/z

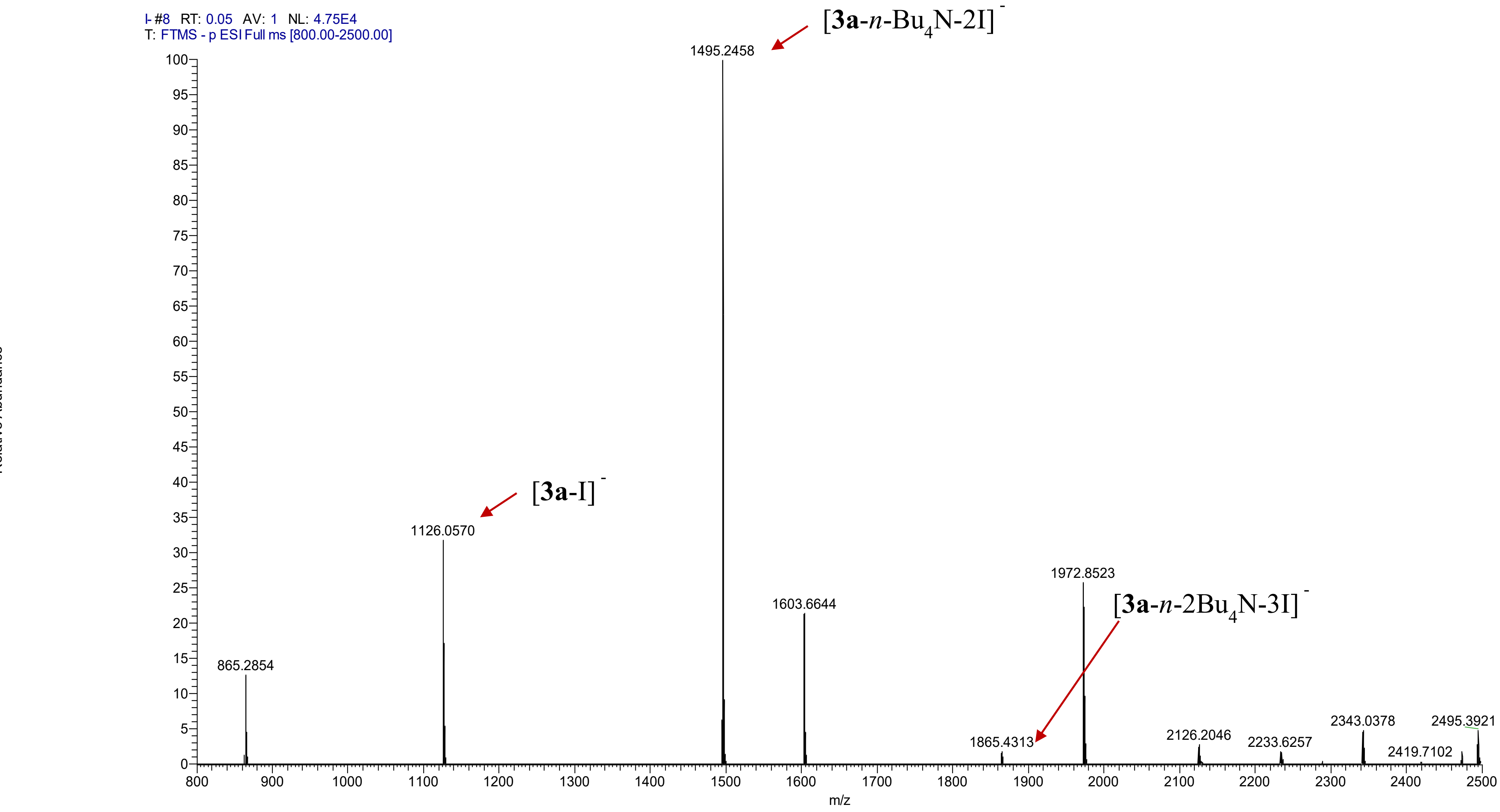

I-#8 RT: 0.05 AV: 1 NL: 4.75E4
T: FTMS - p ESI Full ms [800.00-2500.00]
[3a-n-Bu4N-2I]-
[3a-I]-
[3a-n-2Bu4N-3I]-
1495.2458
1126.0570
1603.6644
1972.8523
865.2854
1865.4313
2126.2046
2233.6257
2343.0378
2419.7102
2495.3921
Relative Abundance
m/z

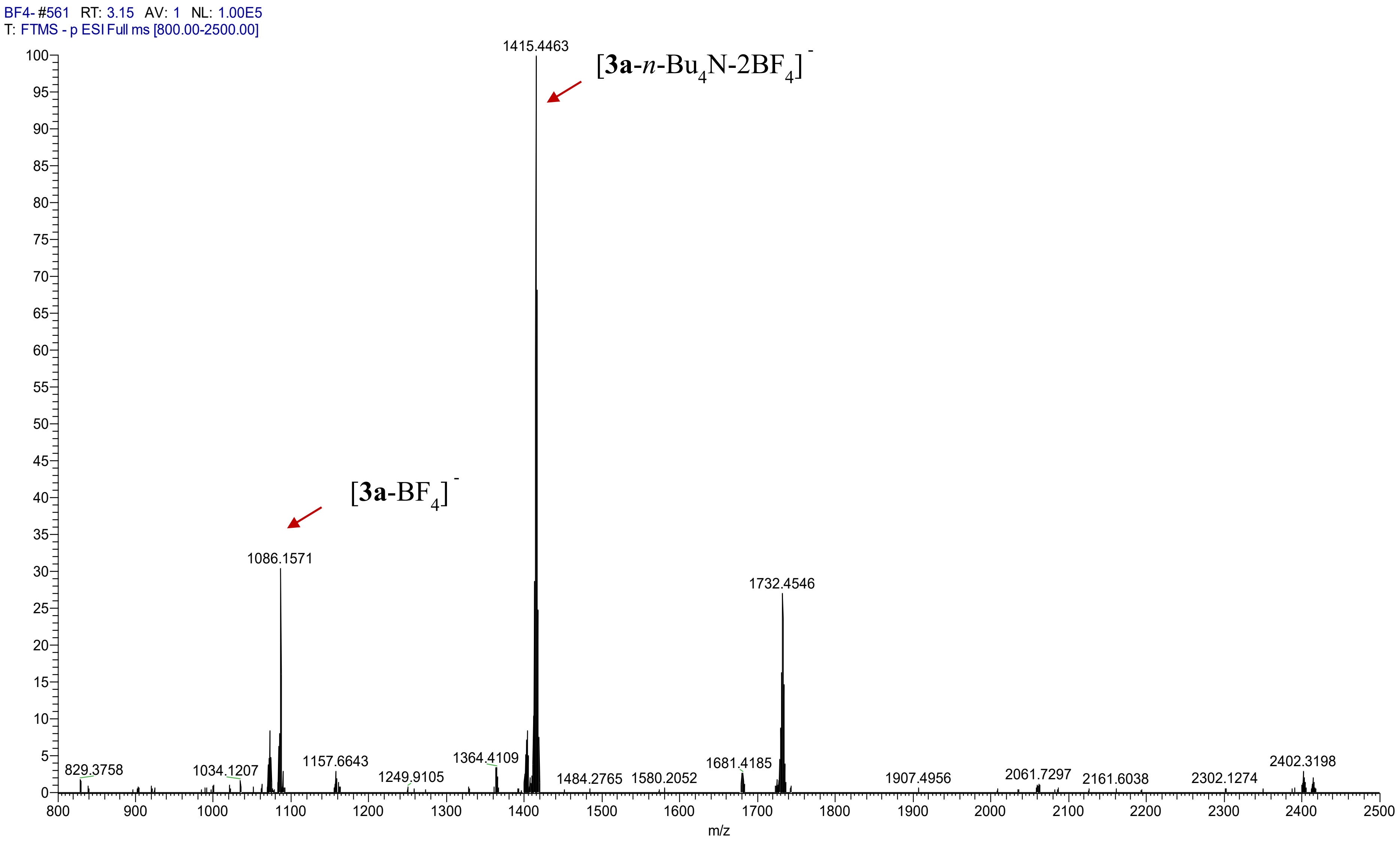

BF4- #561 RT: 3.15 AV: 1 NL: 1.00E5
T: FTMS - p ESI Full ms [800.00-2500.00]
1415.4463
[3a-n-Bu4N-2BF4]-
[3a-BF4]-
1086.1571
1732.4546
829.3758
1034.1207
1157.6643
1249.9105
1364.4109
1484.2765
1580.2052
1681.4185
1907.4956
2061.7297
2161.6038
2302.1274
2402.3198
m/z

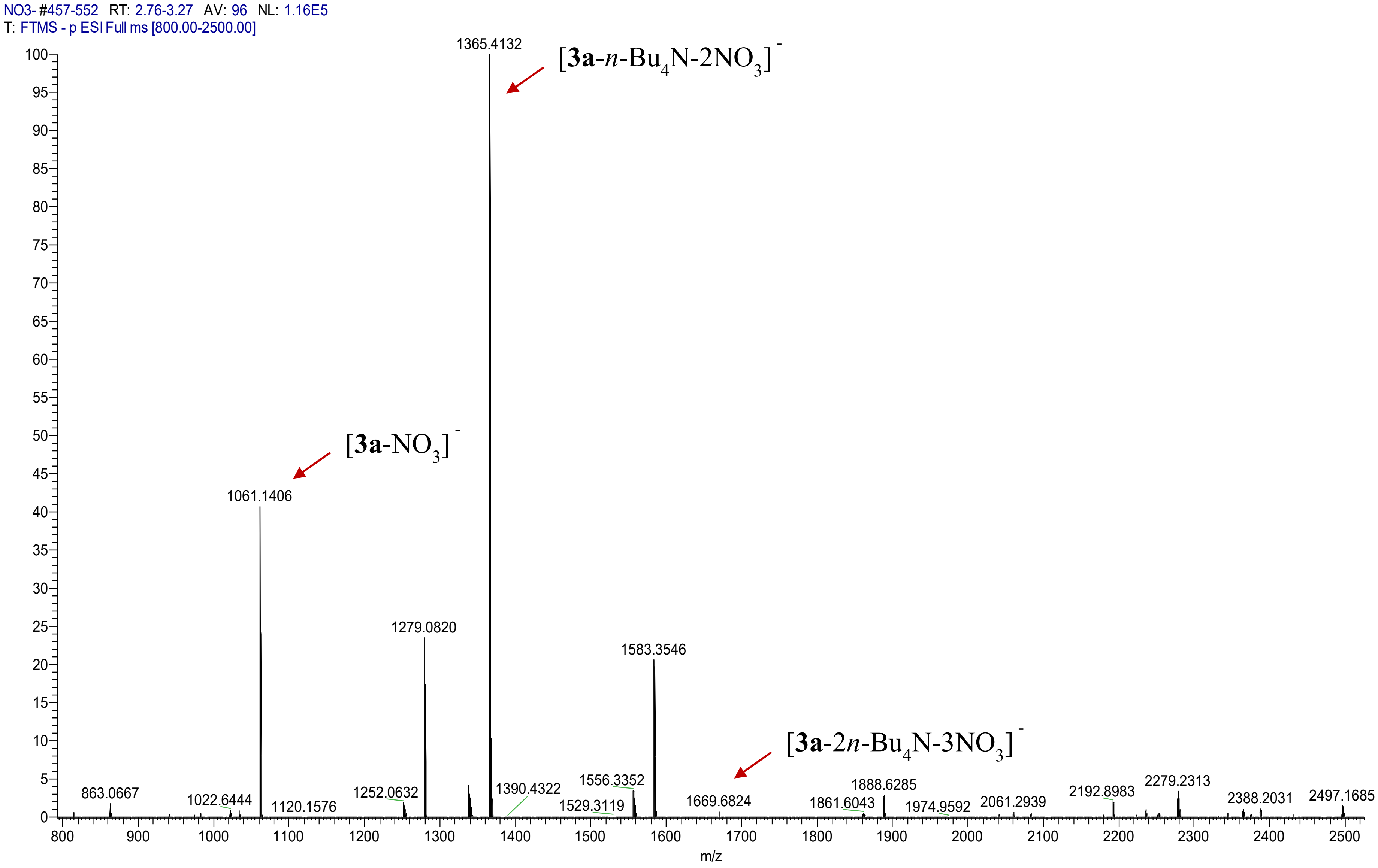

NO3- #457-552 RT: 2.76-3.27 AV: 96 NL: 1.16E5
T: FTMS - p ESI Full ms [800.00-2500.00]
[3a-n-Bu4N-2NO3]-
[3a-NO3]-
[3a-2n-Bu4N-3NO3]-
1365.4132
1061.1406
1279.0820
1583.3546
863.0667
1022.6444
1120.1576
1252.0632
1390.4322
1529.3119
1556.3352
1669.6824
1861.6043
1888.6285
1974.9592
2061.2939
2192.8983
2279.2313
2388.2031
2497.1685
m/z

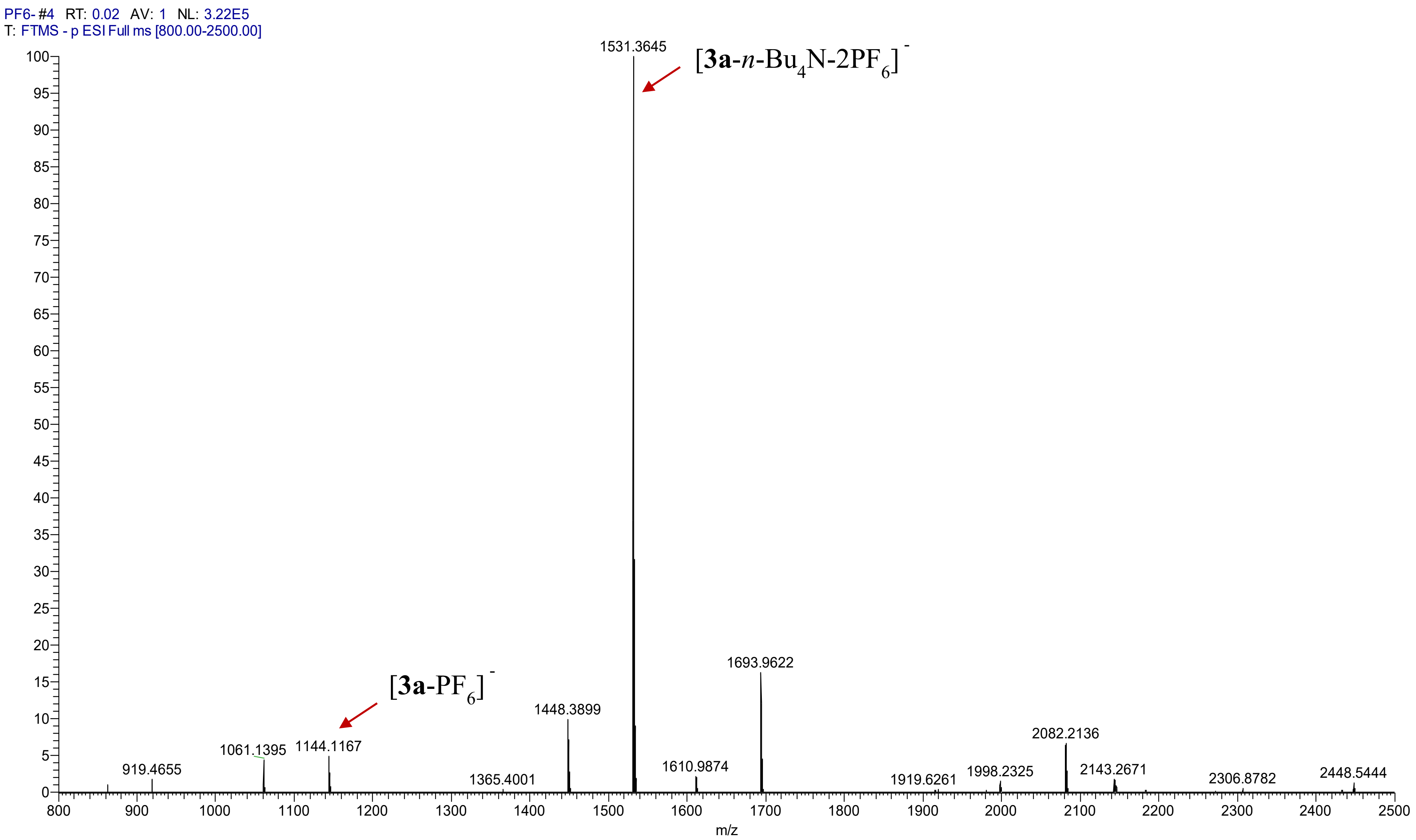

PF6- #4 RT: 0.02 AV: 1 NL: 3.22E5
T: FTMS - p ESI Full ms [800.00-2500.00]
1531.3645
[3a-n-Bu4N-2PF6]-
1693.9622
[3a-PF6]-
1448.3899
2082.2136
1061.1395
1144.1167
919.4655
1610.9874
2143.2671
1365.4001
1919.6261
1998.2325
2306.8782
2448.5444
m/z

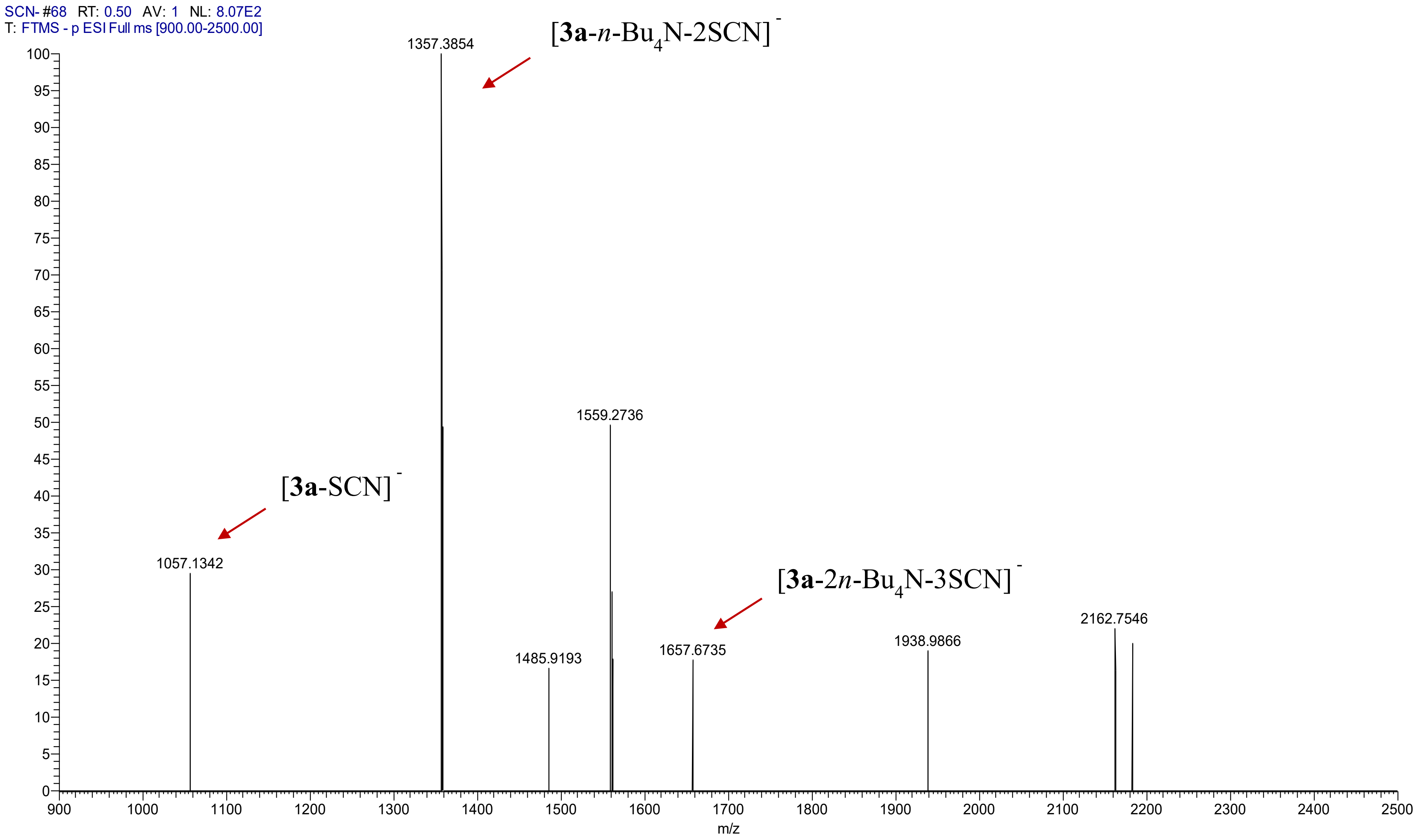

SCN- #68 RT: 0.50 AV: 1 NL: 8.07E2
T: FTMS - p ESI Full ms [900.00-2500.00]
[3a-n-Bu4N-2SCN]-
[3a-SCN]-
[3a-2n-Bu4N-3SCN]-
1357.3854
1559.2736
1057.1342
1485.9193
1657.6735
1938.9866
2162.7546
m/z

5. Copies of ^{1}H and ^{13}C NMR spectra

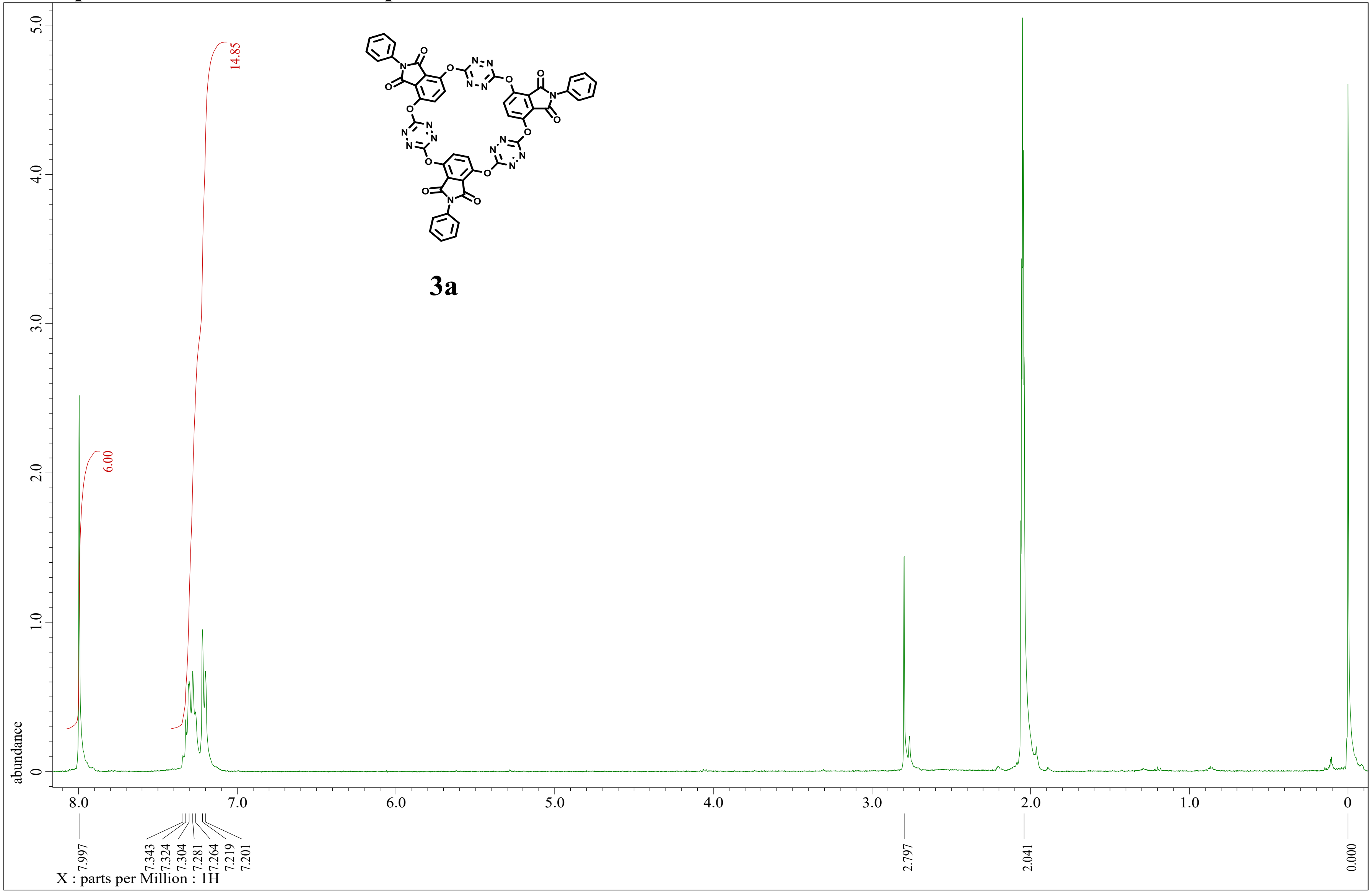

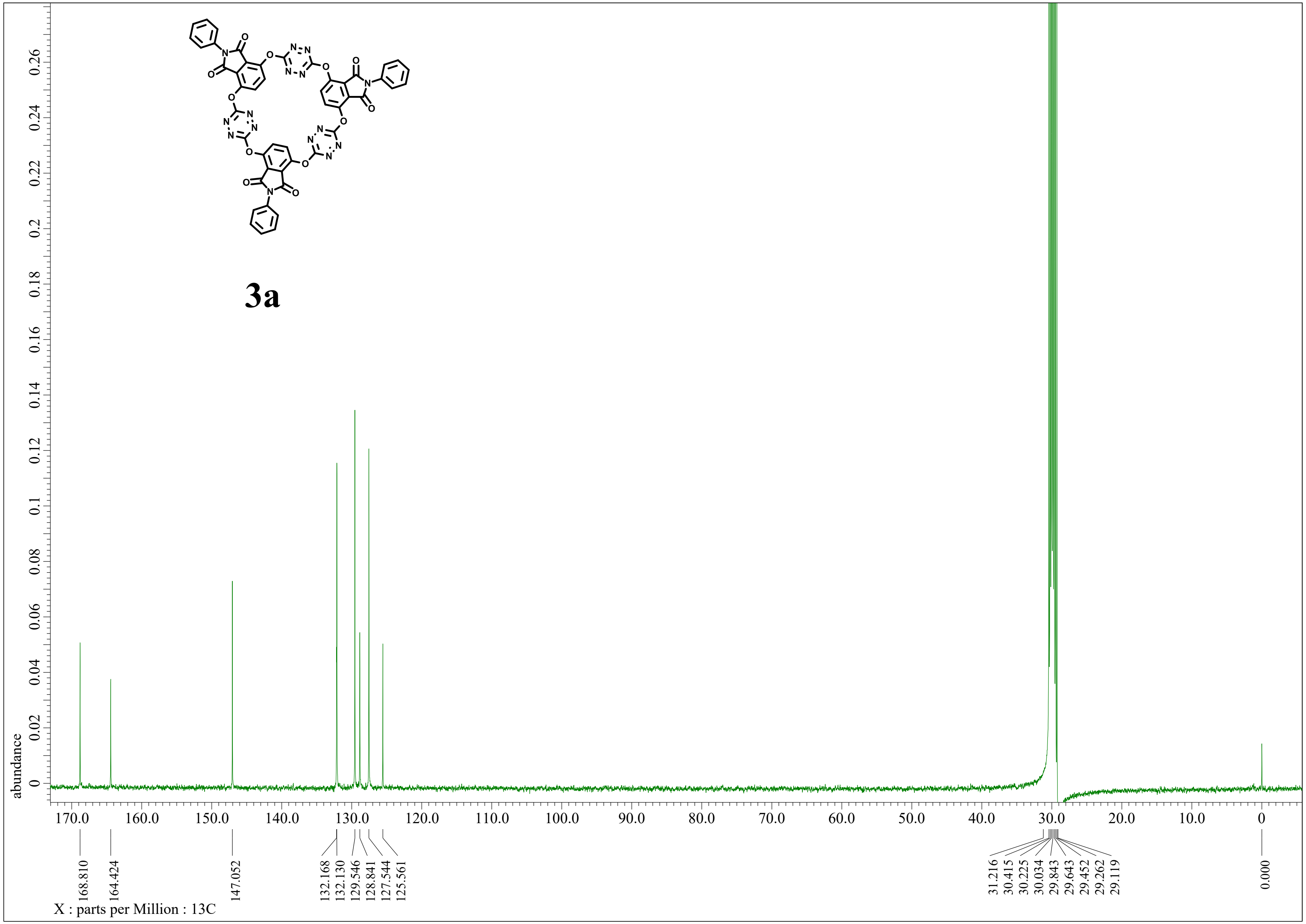
abundance
0.26
0.24
0.22
0.2
0.18
0.16
0.14
0.12
0.1
0.08
0.06
0.04
0.02
0
170.0
160.0
150.0
140.0
130.0
120.0
110.0
100.0
90.0
80.0
70.0
60.0
50.0
40.0
30.0
20.0
10.0
0
168.810
164.424
147.052
132.168
132.130
129.546
128.841
127.544
125.561
31.216
30.415
30.225
30.034
29.843
29.643
29.452
29.262
29.119
0.000
X : parts per Million : 13C

3a

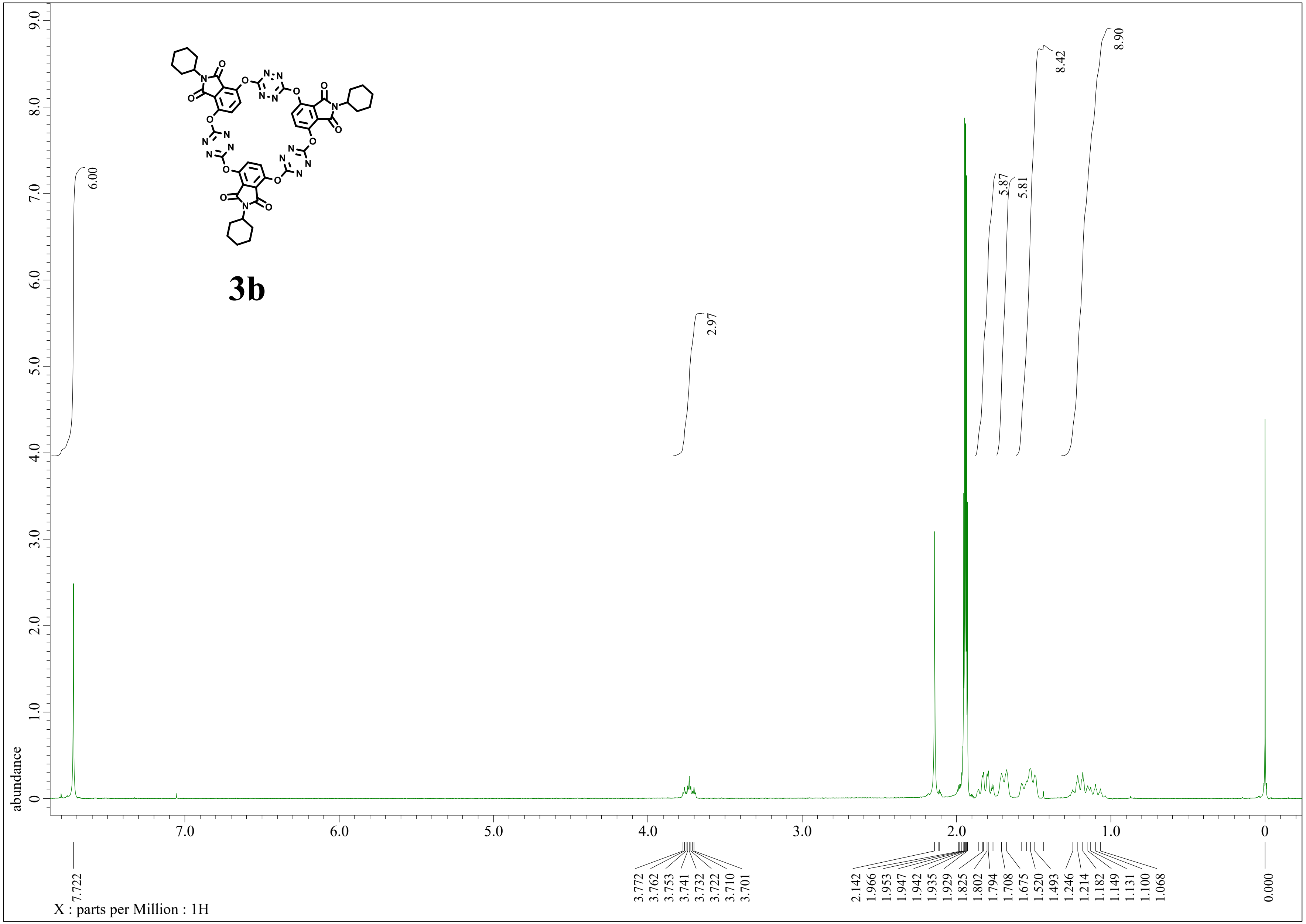
3b
abundance
X : parts per Million : 1H
9.0
8.0
7.0
6.0
5.0
4.0
3.0
2.0
1.0
0
7.0
6.0
5.0
4.0
3.0
2.0
1.0
0
6.00
2.97
5.87
5.81
8.42
8.90
7.722
3.772
3.762
3.753
3.741
3.732
3.722
3.710
3.701
2.142
1.966
1.953
1.947
1.942
1.935
1.929
1.825
1.802
1.794
1.708
1.675
1.520
1.493
1.246
1.214
1.182
1.149
1.131
1.100
1.068
0.000

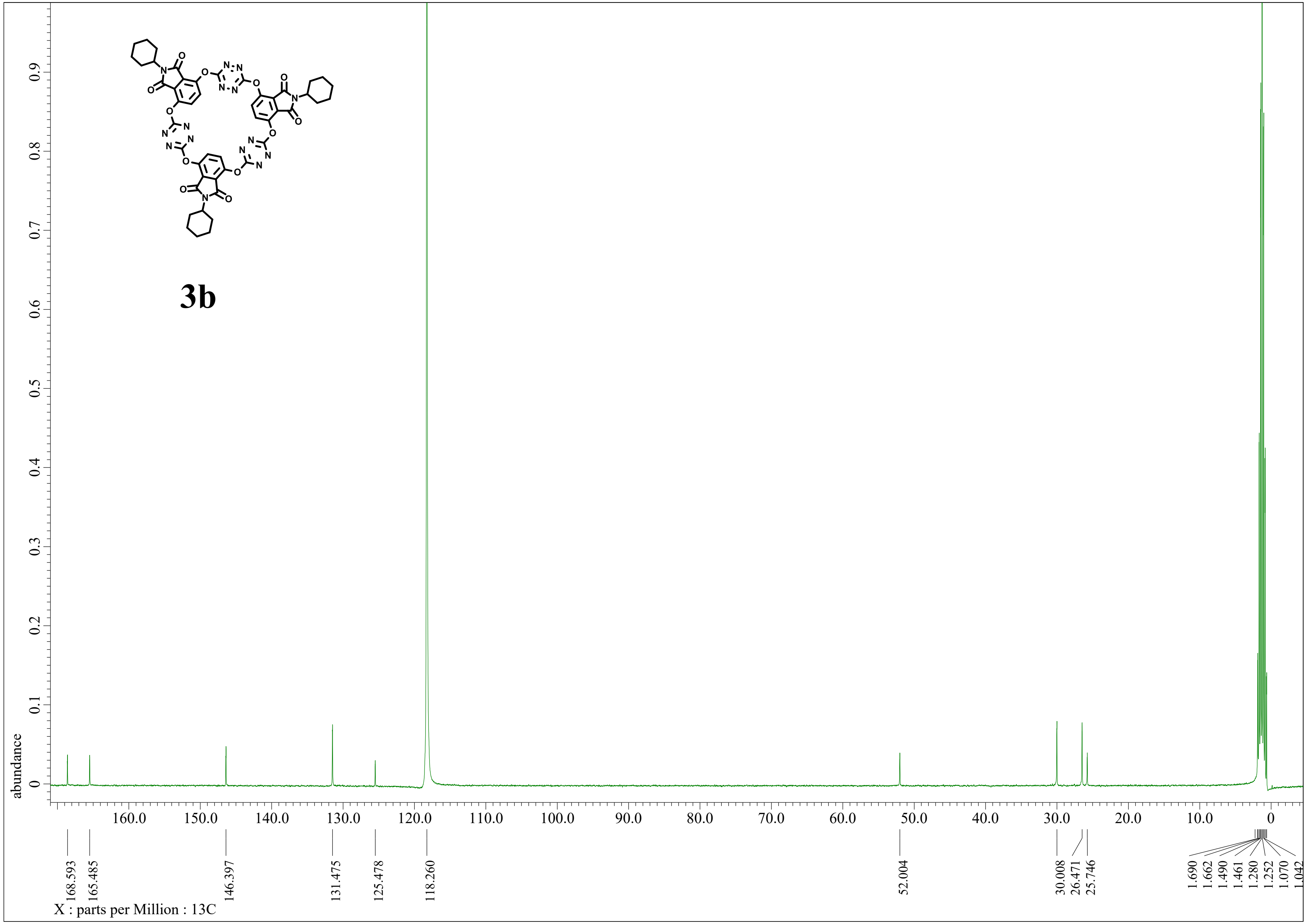
3b
abundance
X : parts per Million : 13C
168.593
165.485
146.397
131.475
125.478
118.260
52.004
30.008
26.471
25.746
1.690
1.662
1.490
1.461
1.280
1.252
1.070
1.042

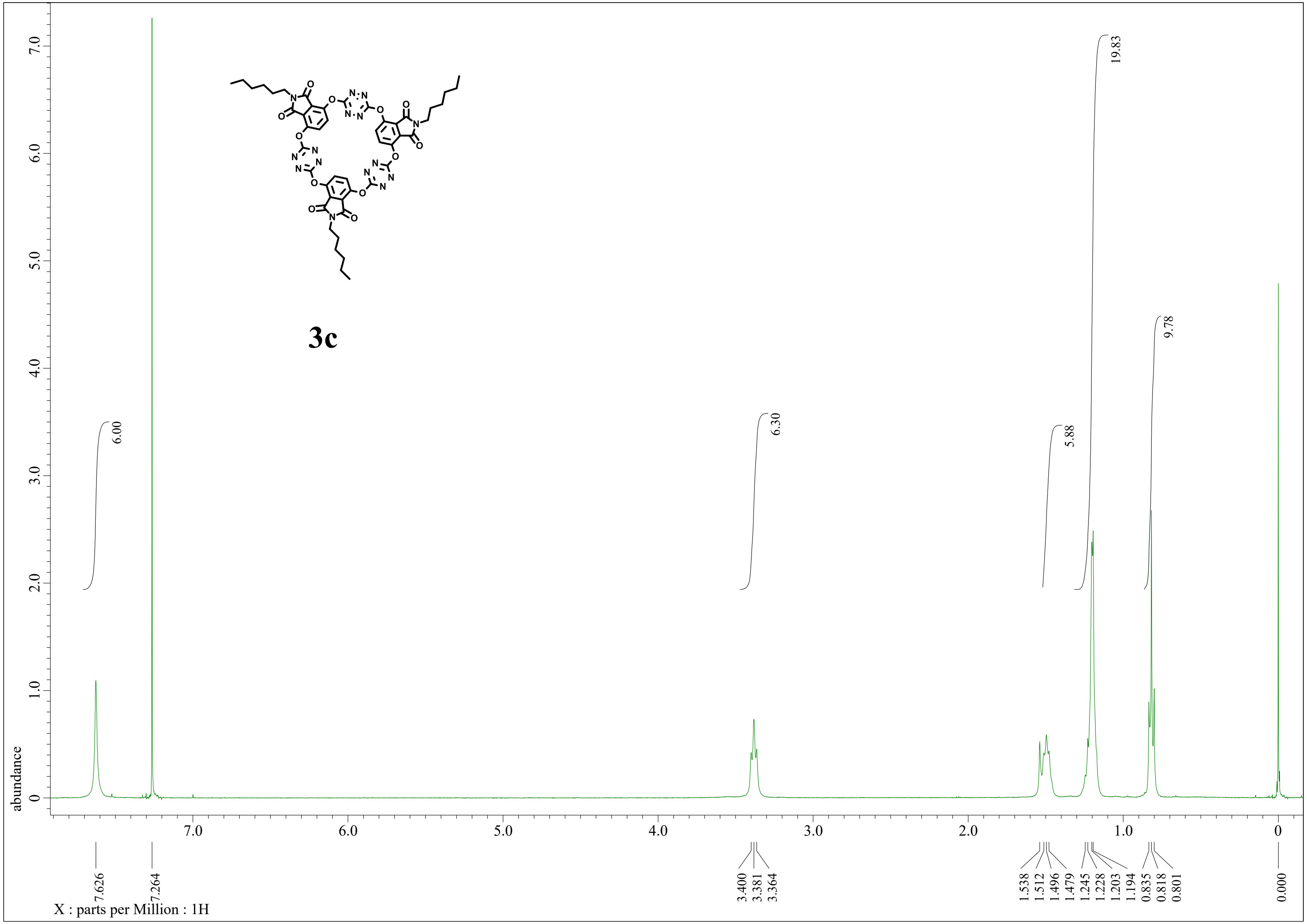
3c
abundance
X : parts per Million : 1H
6.00
6.30
5.88
19.83
9.78
7.626
7.264
3.400
3.381
3.364
1.538
1.512
1.496
1.479
1.245
1.228
1.203
1.194
0.835
0.818
0.801
0.000

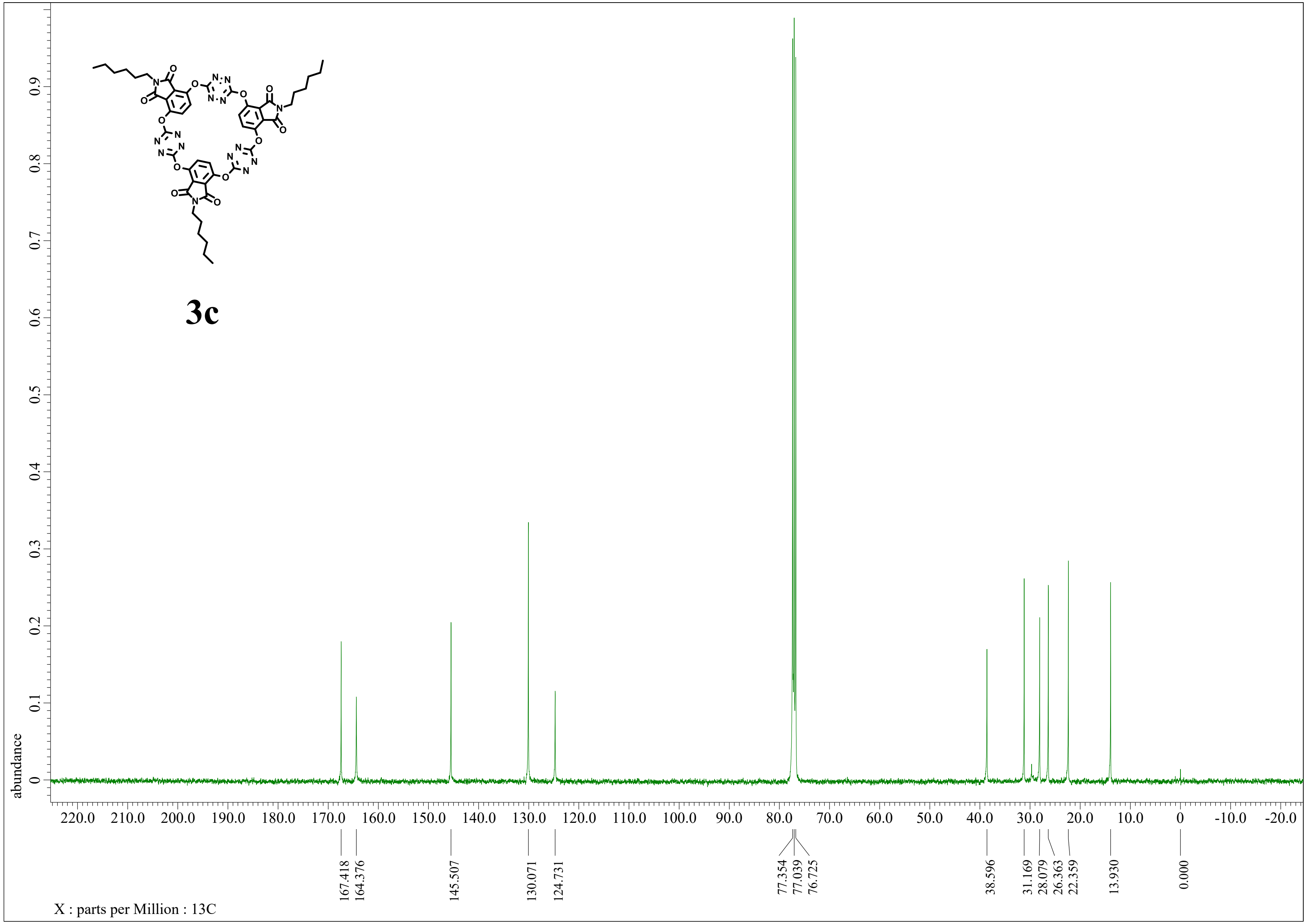
3c
abundance
0
0.1
0.2
0.3
0.4
0.5
0.6
0.7
0.8
0.9
220.0 210.0 200.0 190.0 180.0 170.0 160.0 150.0 140.0 130.0 120.0 110.0 100.0 90.0 80.0 70.0 60.0 50.0 40.0 30.0 20.0 10.0 0 -10.0 -20.0
167.418
164.376
145.507
130.071
124.731
77.354
77.039
76.725
38.596
31.169
28.079
26.363
22.359
13.930
0.000
X : parts per Million : 13C

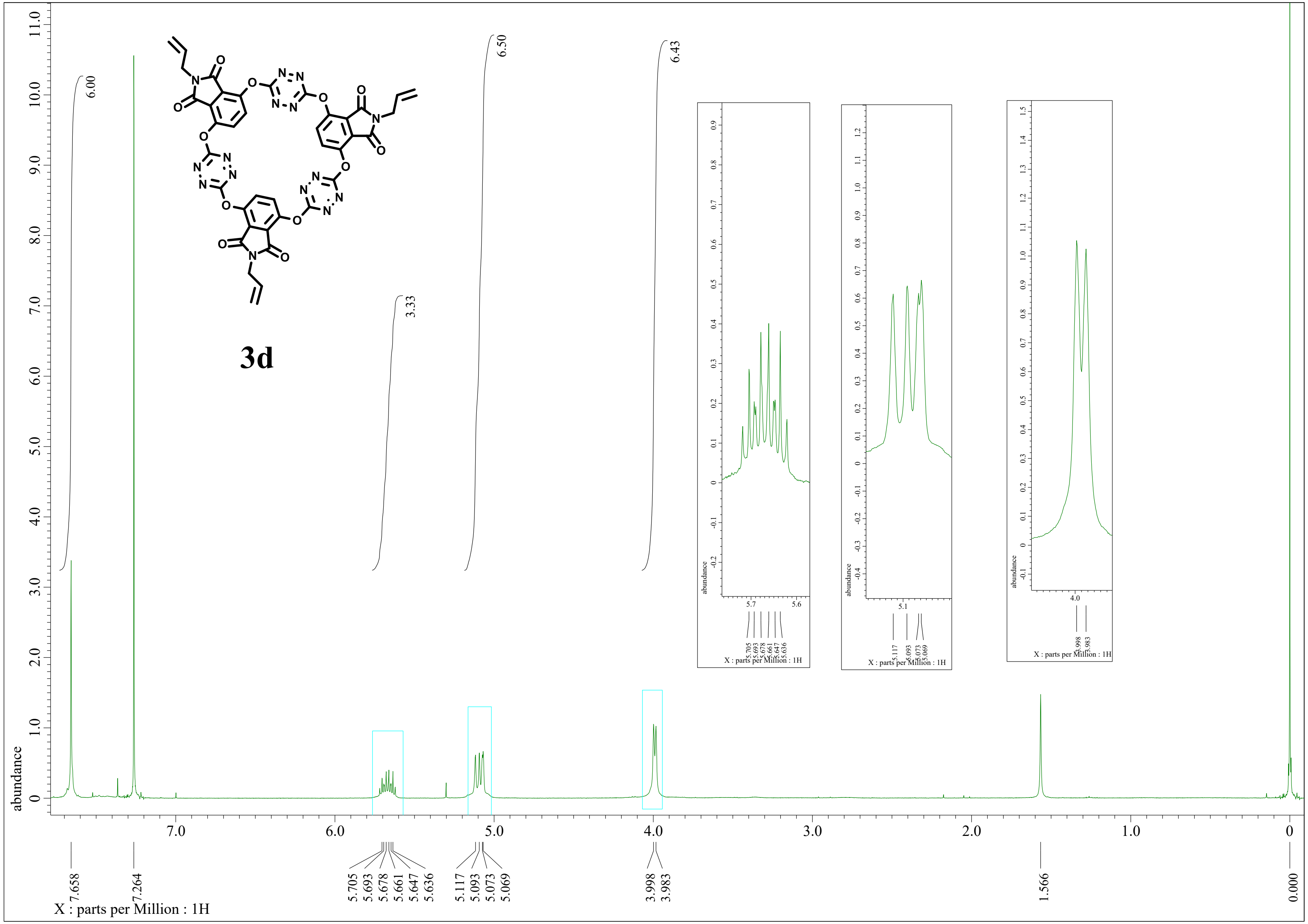
3d
6.00
3.33
6.50
6.43
abundance
X : parts per Million : 1H
7.658
7.264
5.705
5.693
5.678
5.661
5.647
5.636
5.117
5.093
5.073
5.069
3.998
3.983
1.566
0.000

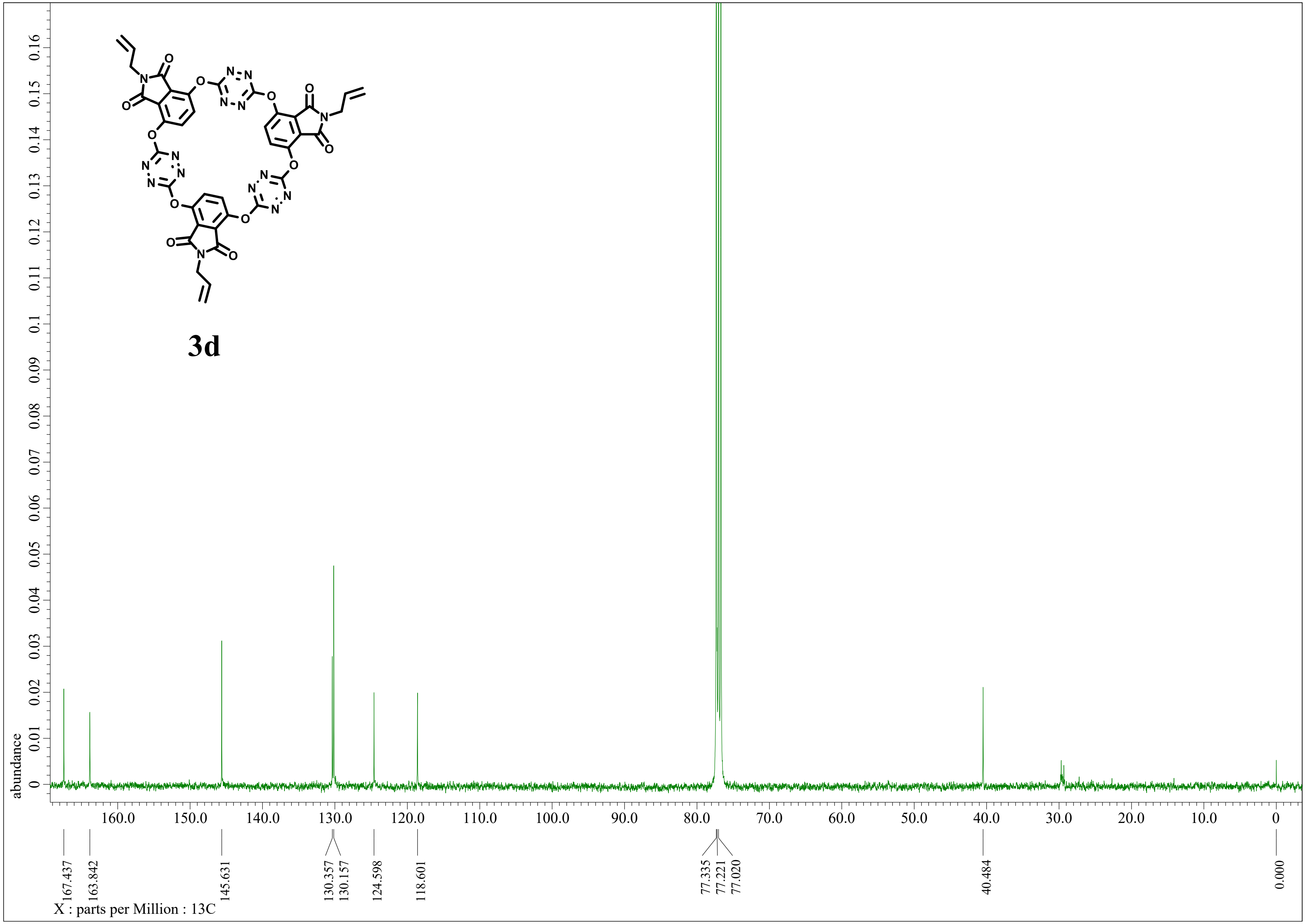
3d
abundance
X : parts per Million : 13C
167.437
163.842
145.631
130.357
130.157
124.598
118.601
77.335
77.221
77.020
40.484
0.000

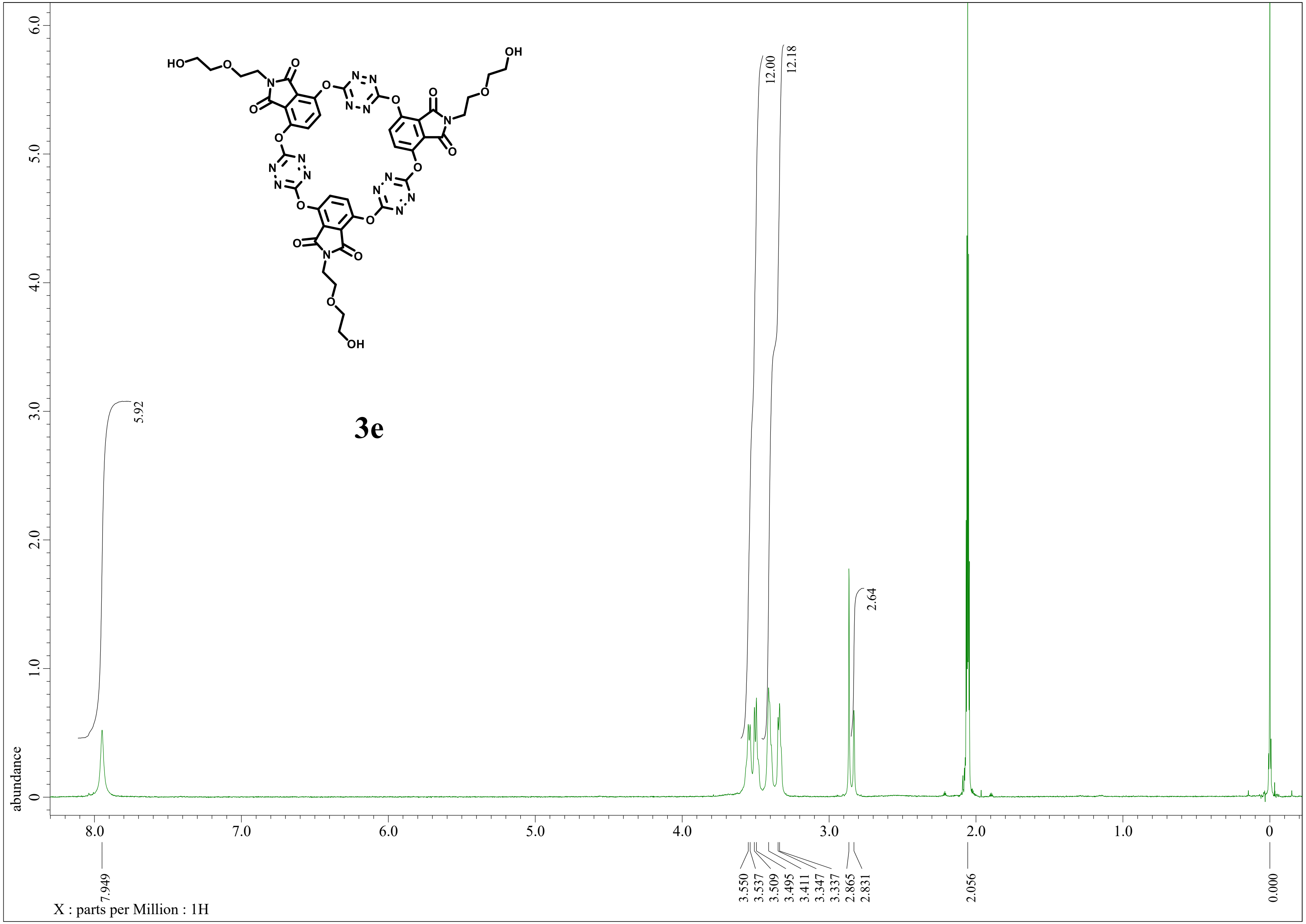

3e
HO
OH
O
N
abundance
X : parts per Million : 1H
5.92
12.00
12.18
2.64
7.949
3.550
3.537
3.509
3.495
3.411
3.347
3.337
2.865
2.831
2.056
0.000

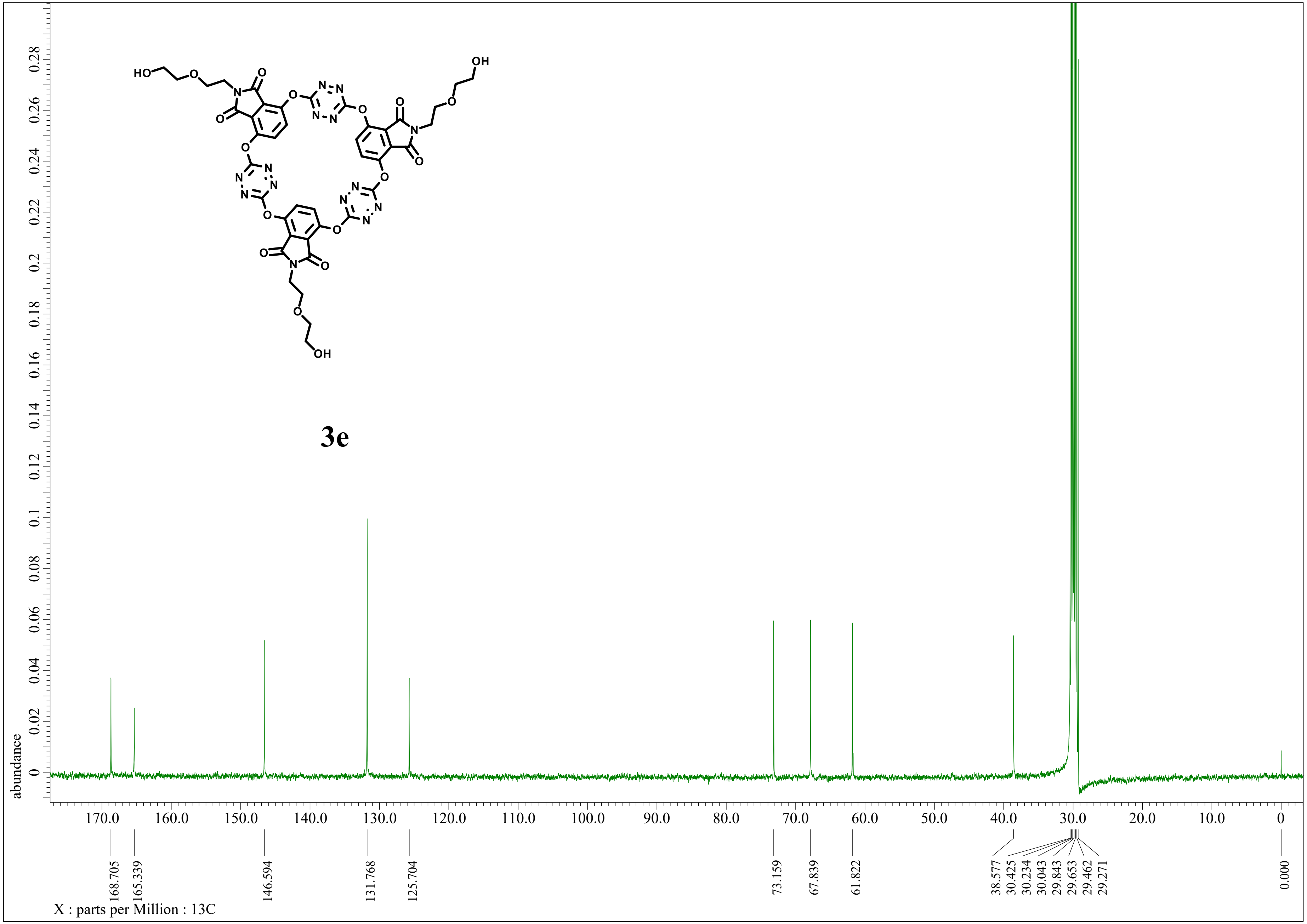

HO
O
N
O
O
OH
3e
abundance
0.28
0.26
0.24
0.22
0.2
0.18
0.16
0.14
0.12
0.1
0.08
0.06
0.04
0.02
0
170.0
160.0
150.0
140.0
130.0
120.0
110.0
100.0
90.0
80.0
70.0
60.0
50.0
40.0
30.0
20.0
10.0
0
168.705
165.339
146.594
131.768
125.704
73.159
67.839
61.822
38.577
30.425
30.234
30.043
29.843
29.653
29.462
29.271
0.000
X : parts per Million : 13C

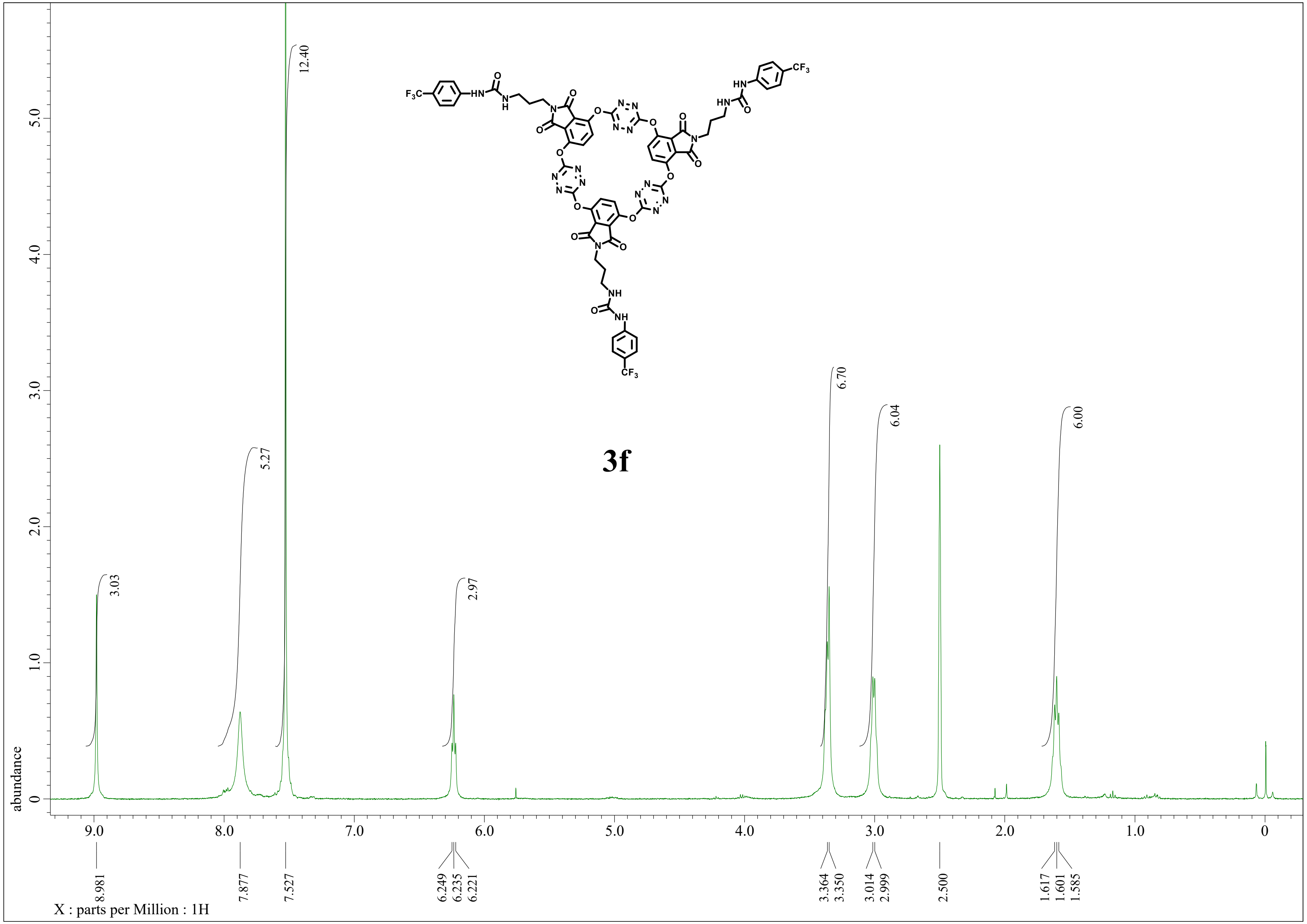
3f
F3C
HN
H
N
O
CF3
NH
abundance
0
1.0
2.0
3.0
4.0
5.0
3.03
5.27
12.40
2.97
6.70
6.04
6.00
9.0
8.0
7.0
6.0
5.0
4.0
3.0
2.0
1.0
0
8.981
7.877
7.527
6.249
6.235
6.221
3.364
3.350
3.014
2.999
2.500
1.617
1.601
1.585
X : parts per Million : 1H

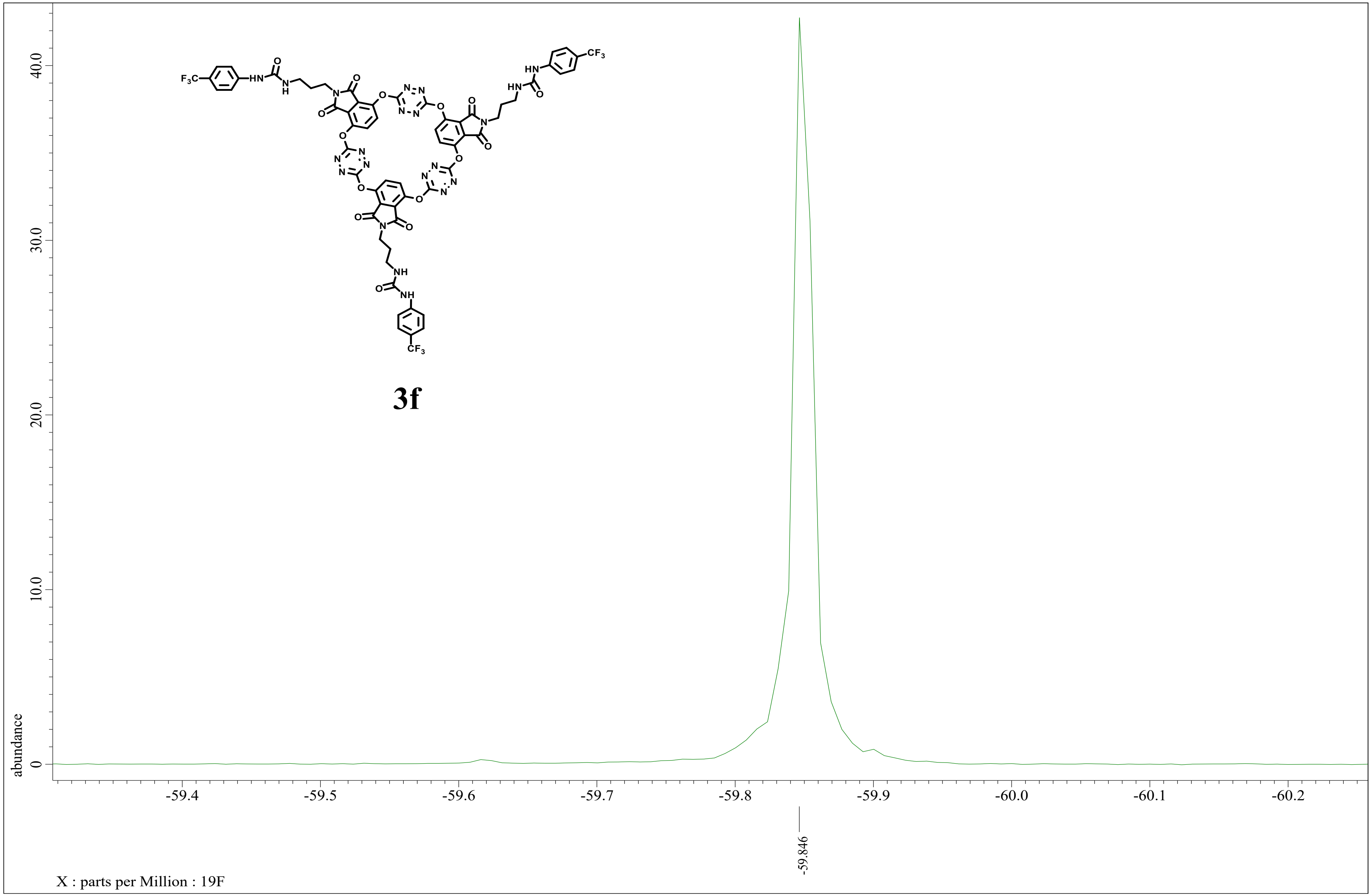
3f
40.0
30.0
20.0
10.0
0
abundance
-59.4
-59.5
-59.6
-59.7
-59.8
-59.9
-60.0
-60.1
-60.2
-59.846
X : parts per Million : 19F

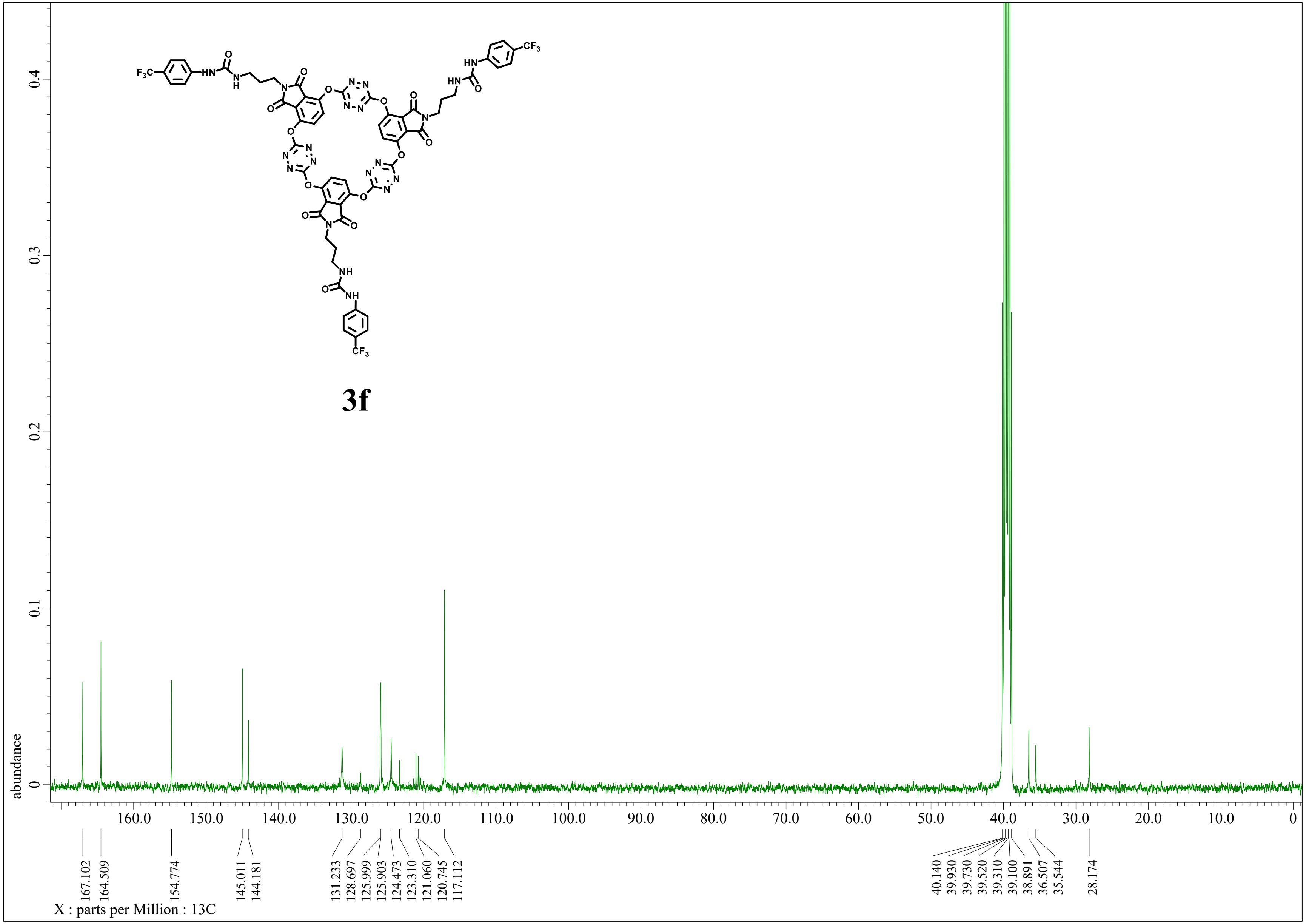

3f
abundance
0
0.1
0.2
0.3
0.4
160.0
150.0
140.0
130.0
120.0
110.0
100.0
90.0
80.0
70.0
60.0
50.0
40.0
30.0
20.0
10.0
0
167.102
164.509
154.774
145.011
144.181
131.233
128.697
125.999
125.903
124.473
123.310
121.060
120.745
117.112
40.140
39.930
39.730
39.520
39.310
39.100
38.891
36.507
35.544
28.174
X : parts per Million : 13C

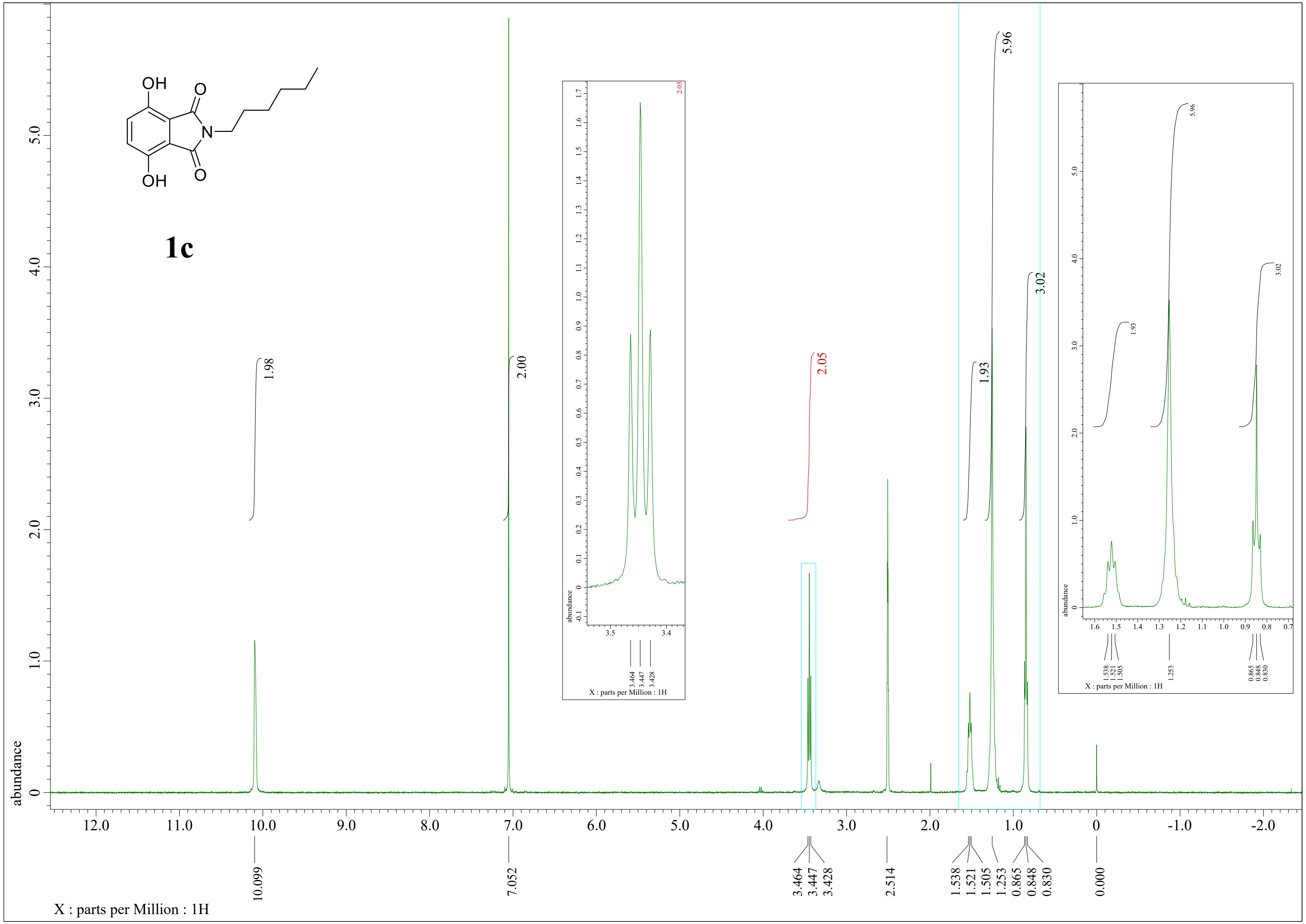
1c
abundance
X : parts per Million : 1H
1.98
2.00
2.05
1.93
5.96
3.02
10.099
7.052
3.464
3.447
3.428
2.514
1.538
1.521
1.505
1.253
0.865
0.848
0.830
0.000

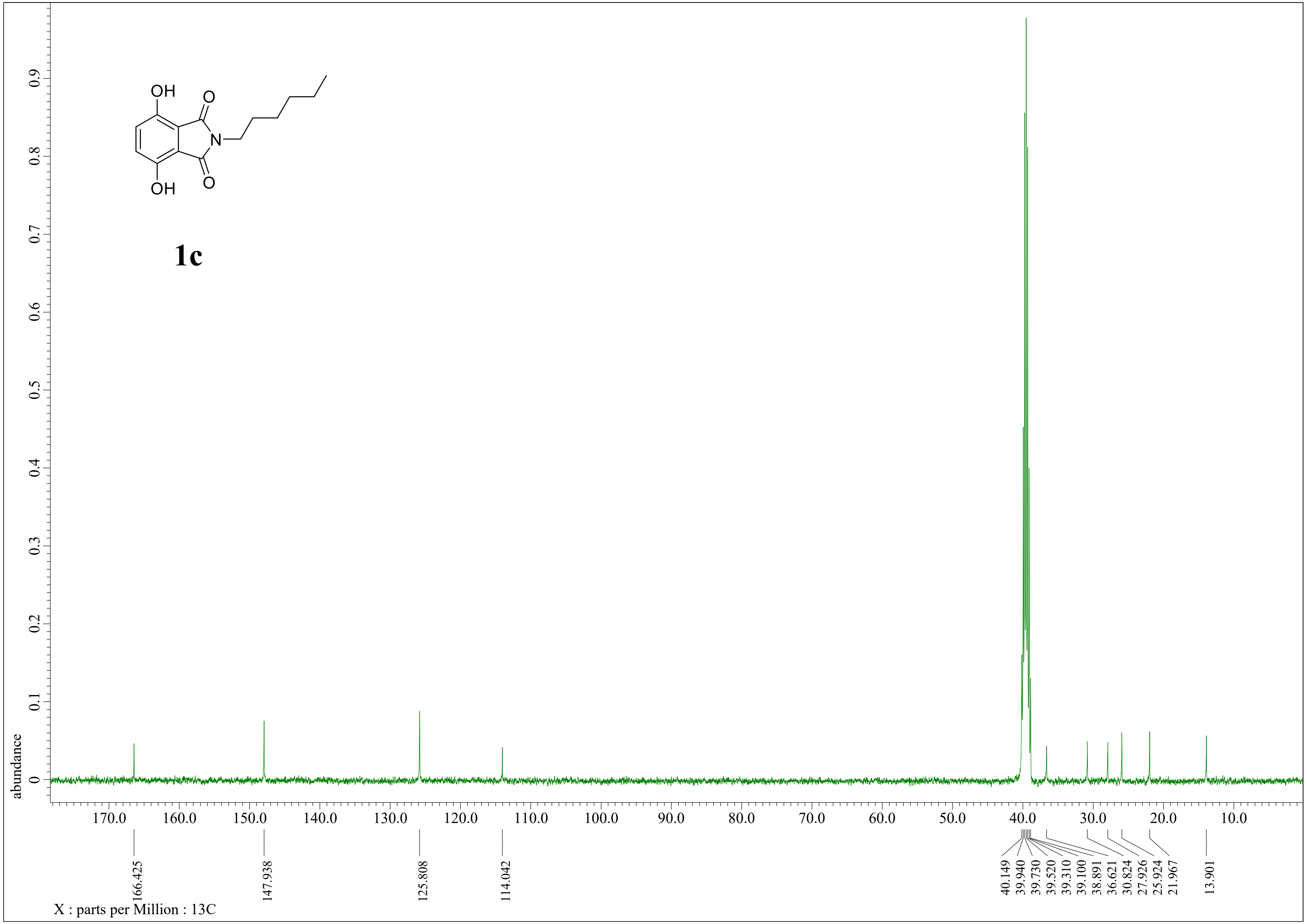
OH
O
N
O
OH
1c
abundance
0
0.1
0.2
0.3
0.4
0.5
0.6
0.7
0.8
0.9
170.0
160.0
150.0
140.0
130.0
120.0
110.0
100.0
90.0
80.0
70.0
60.0
50.0
40.0
30.0
20.0
10.0
166.425
147.938
125.808
114.042
40.149
39.940
39.730
39.520
39.310
39.100
38.891
36.621
30.824
27.926
25.924
21.967
13.901
X : parts per Million : 13C

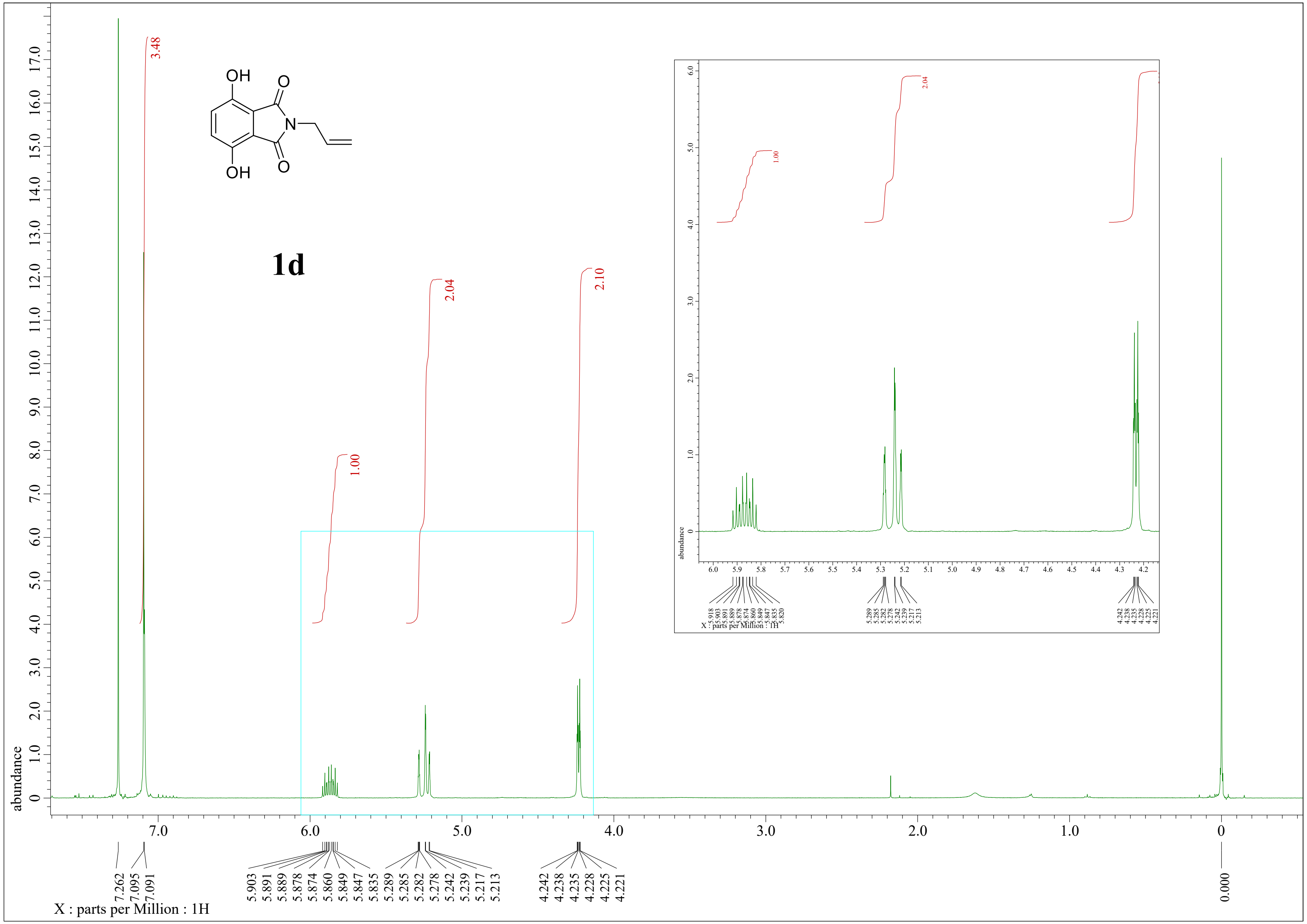
OH
O
N
OH
O
1d
3.48
1.00
2.04
2.10
abundance
X : parts per Million : 1H
7.262
7.095
7.091
5.903
5.891
5.889
5.878
5.874
5.860
5.849
5.847
5.835
5.289
5.285
5.282
5.278
5.242
5.239
5.217
5.213
4.242
4.238
4.235
4.228
4.225
4.221
0.000

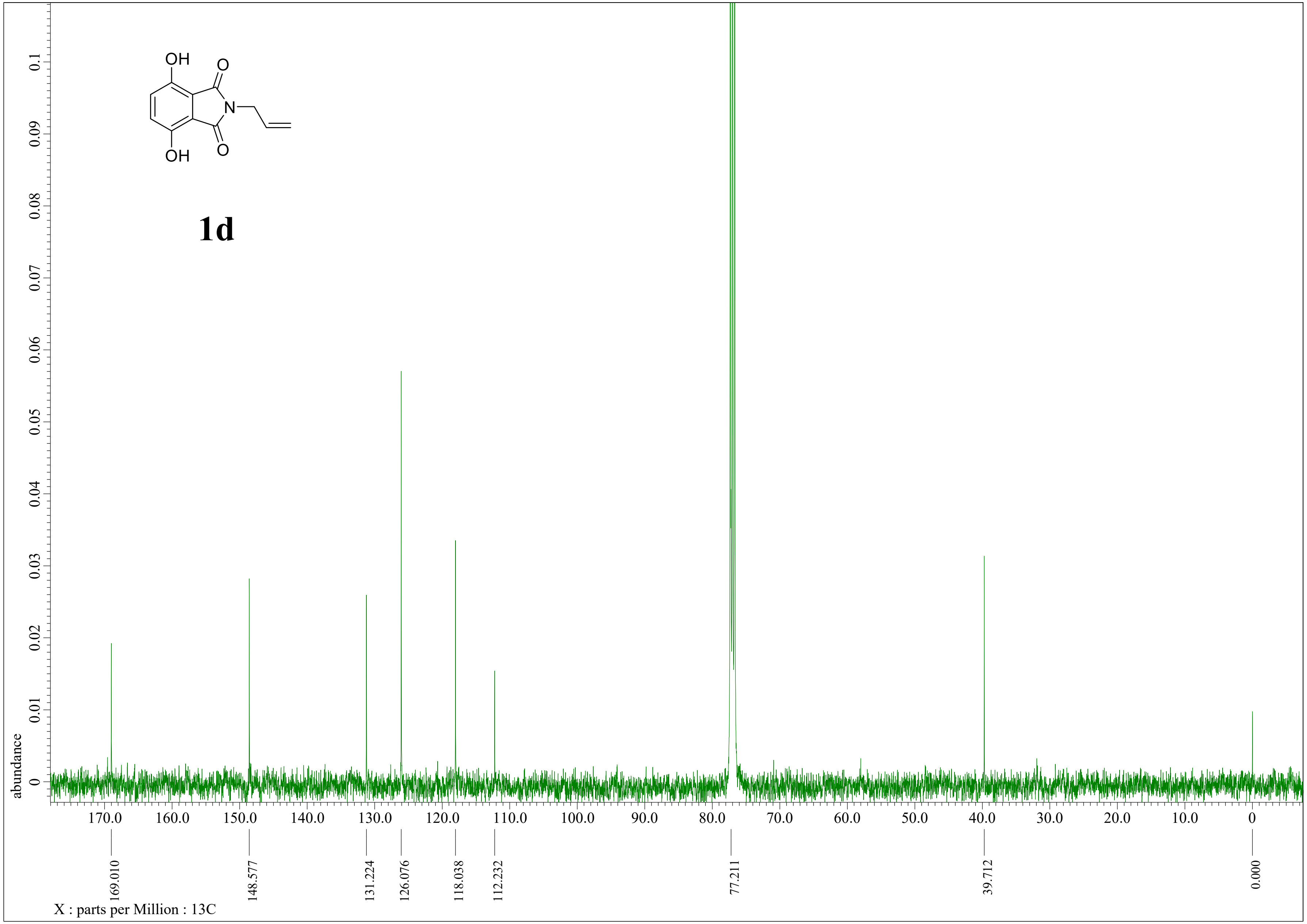
OH
O
N
OH
O
1d
abundance
0.1
0.09
0.08
0.07
0.06
0.05
0.04
0.03
0.02
0.01
0
170.0
160.0
150.0
140.0
130.0
120.0
110.0
100.0
90.0
80.0
70.0
60.0
50.0
40.0
30.0
20.0
10.0
0
169.010
148.577
131.224
126.076
118.038
112.232
77.211
39.712
0.000
X : parts per Million : 13C

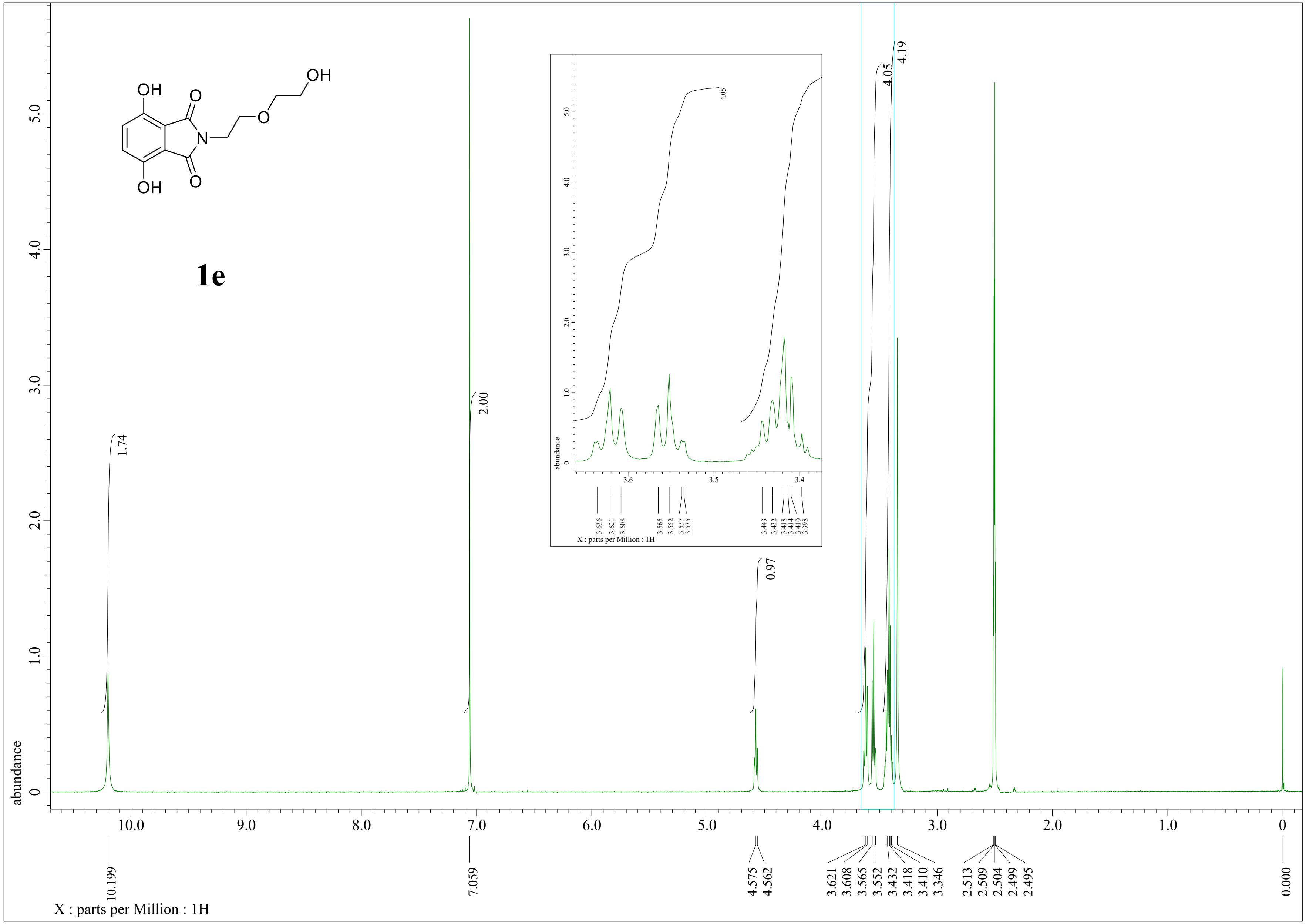
1e
OH
OH
O
O
N
O
OH
abundance
X : parts per Million : 1H
10.0
9.0
8.0
7.0
6.0
5.0
4.0
3.0
2.0
1.0
0
1.74
2.00
0.97
4.05
4.19
10.199
7.059
4.575
4.562
3.621
3.608
3.565
3.552
3.432
3.418
3.410
3.346
2.513
2.509
2.504
2.499
2.495
0.000
3.636
3.621
3.608
3.565
3.552
3.537
3.535
3.443
3.432
3.418
3.414
3.410
3.398

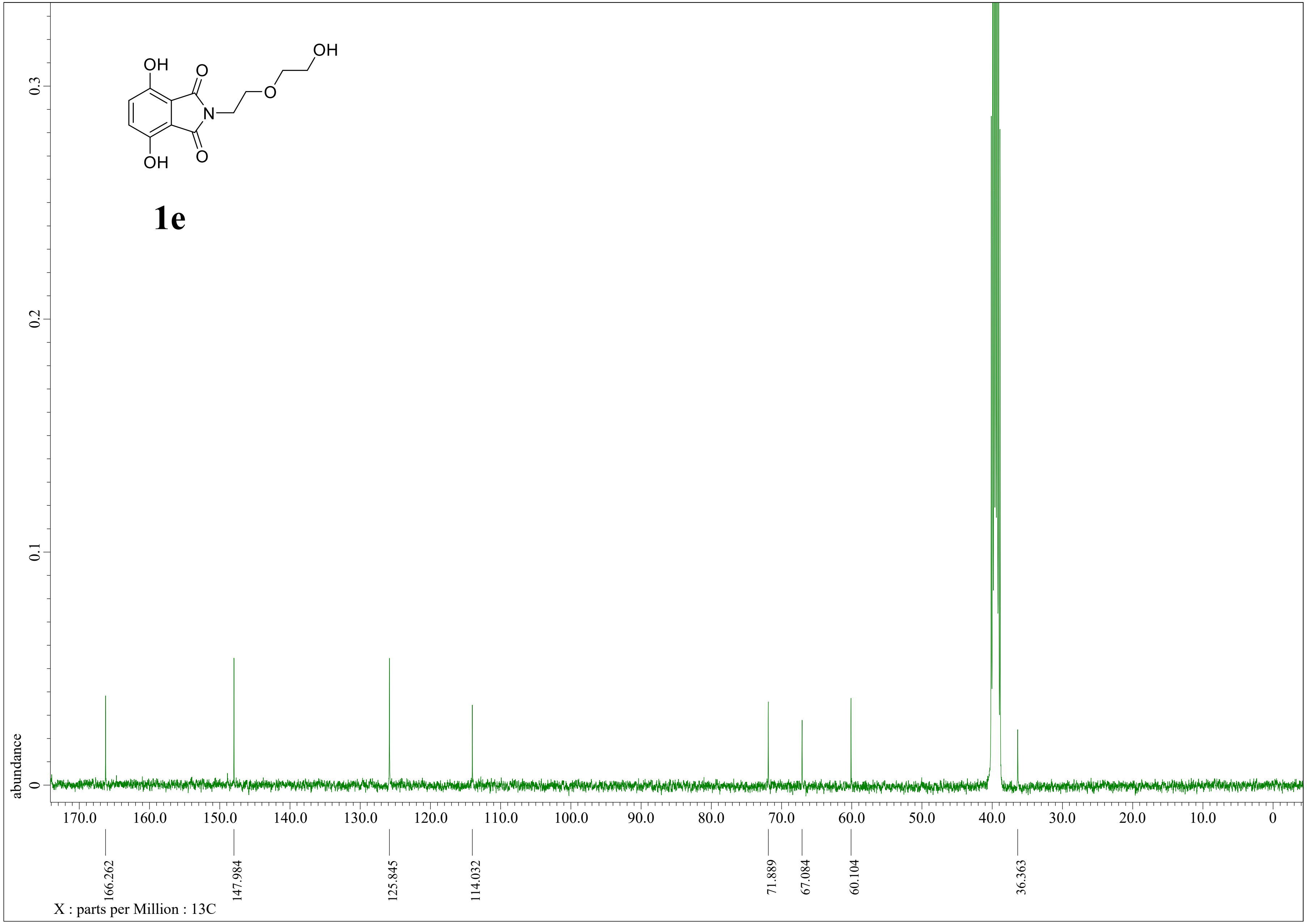
OH
O
OH
N
O
OH
O
1e
abundance
0
0.1
0.2
0.3
170.0 160.0 150.0 140.0 130.0 120.0 110.0 100.0 90.0 80.0 70.0 60.0 50.0 40.0 30.0 20.0 10.0 0
166.262
147.984
125.845
114.032
71.889
67.084
60.104
36.363
X : parts per Million : 13C

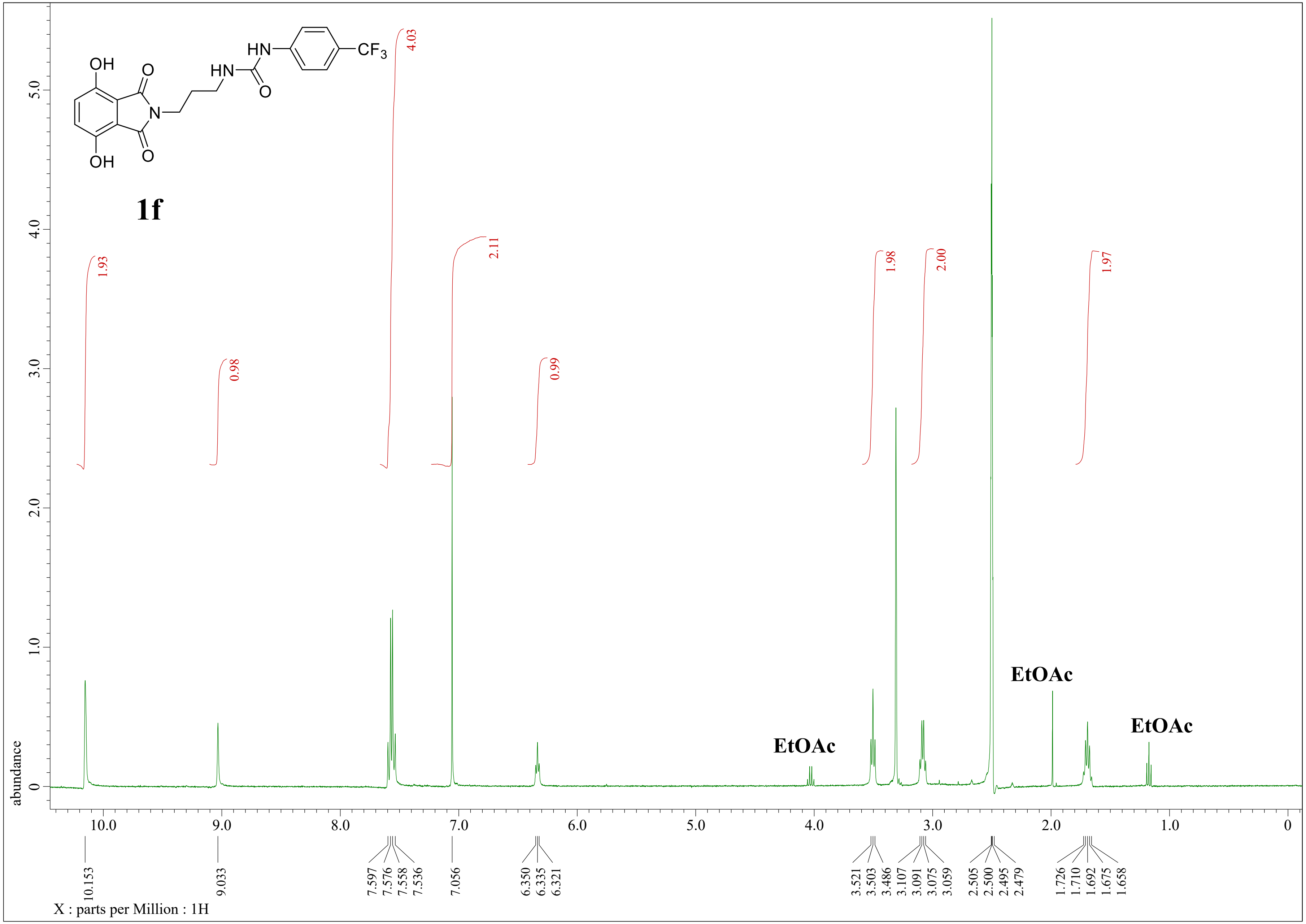

OH
O
HN
HN
CF3
N
O
OH
O
1f
1.93
0.98
4.03
2.11
0.99
1.98
2.00
1.97
EtOAc
EtOAc
EtOAc
abundance
X : parts per Million : 1H
10.153
9.033
7.597
7.576
7.558
7.536
7.056
6.350
6.335
6.321
3.521
3.503
3.486
3.107
3.091
3.075
3.059
2.505
2.500
2.495
2.479
1.726
1.710
1.692
1.675
1.658

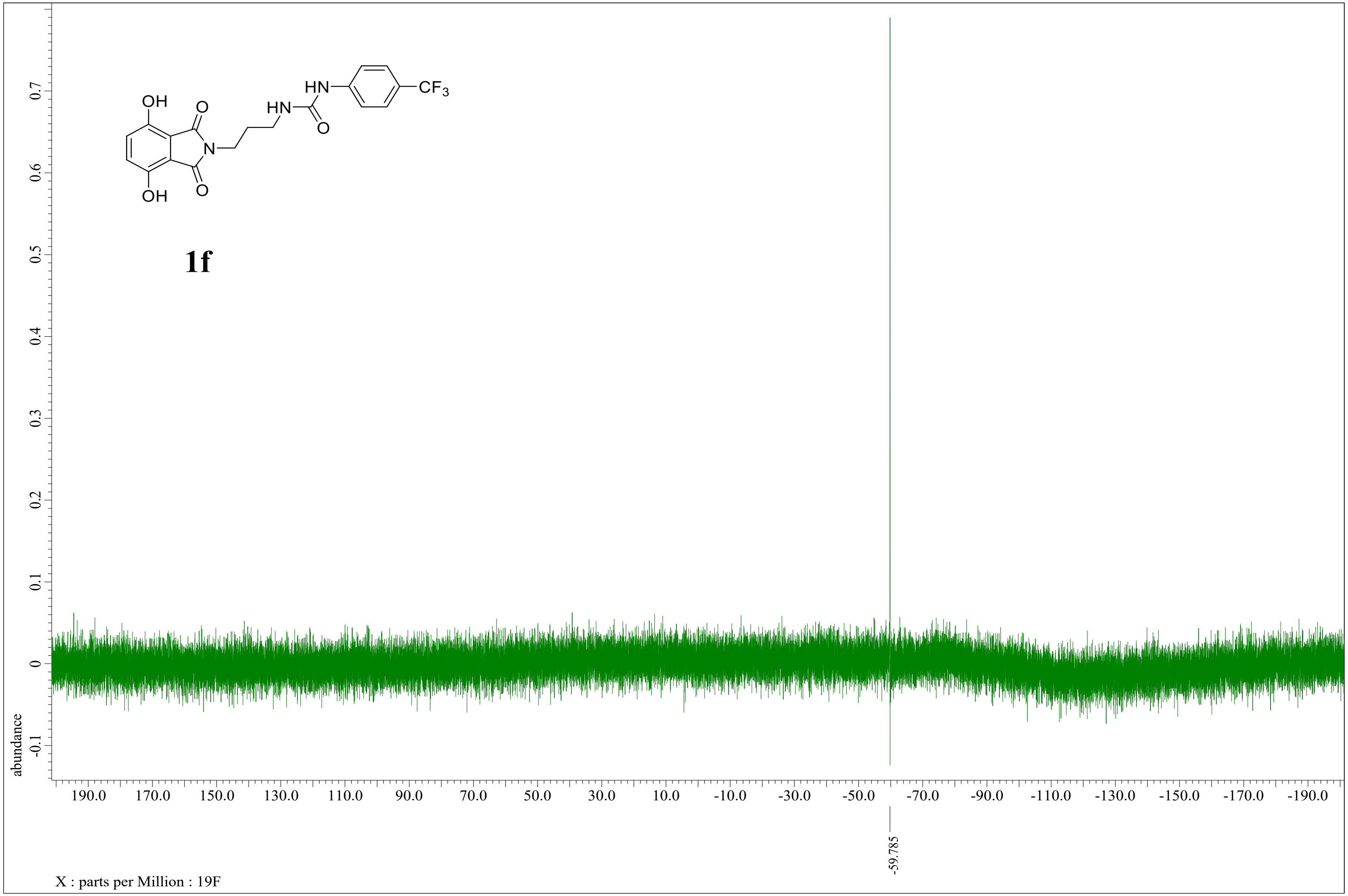
1f
OH
OH
O
O
N
HN
HN
O
CF3
-59.785
abundance
X : parts per Million : 19F

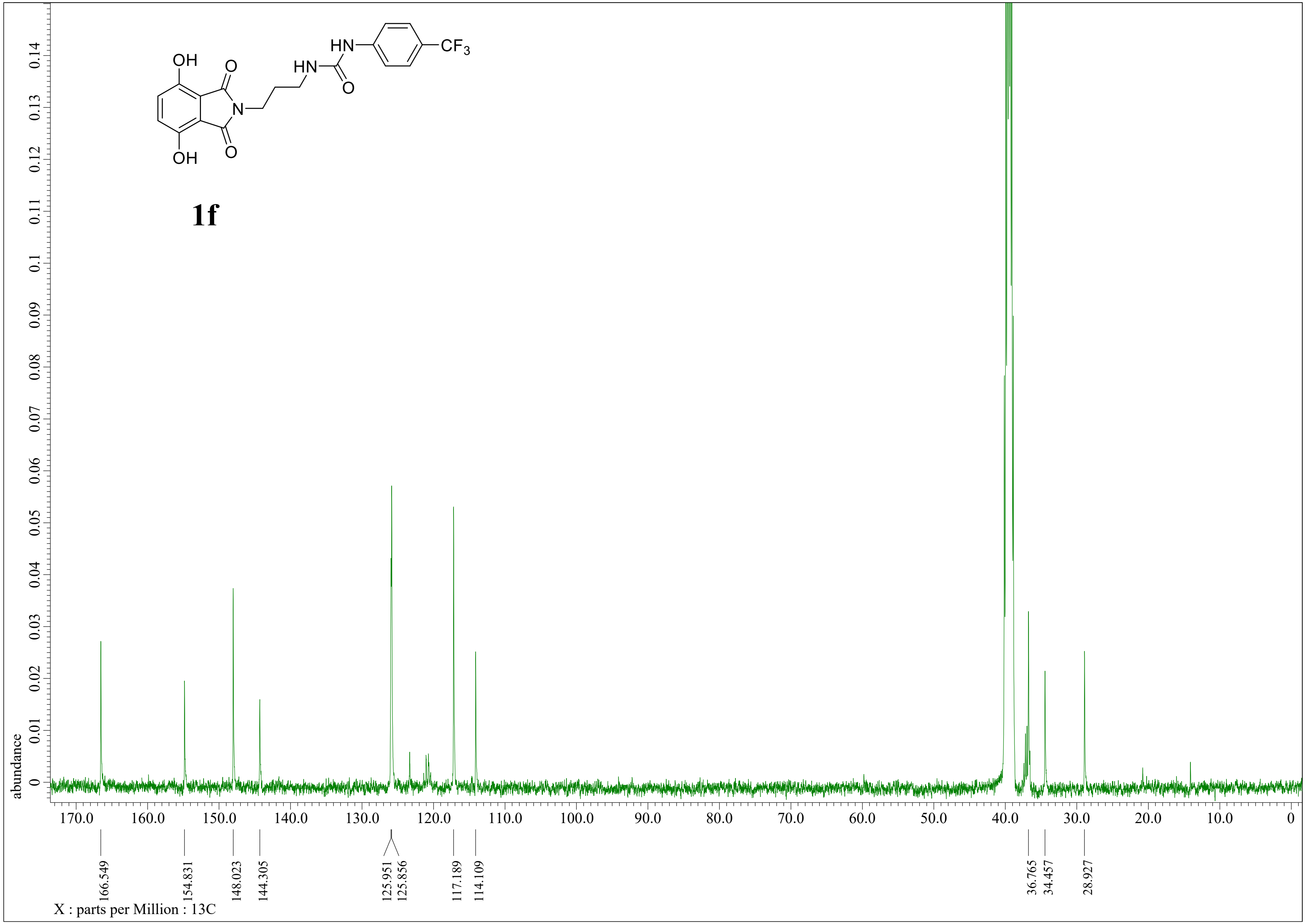

OH
O
HN
HN
CF3
O
N
O
OH
1f
abundance
0
0.01
0.02
0.03
0.04
0.05
0.06
0.07
0.08
0.09
0.1
0.11
0.12
0.13
0.14
170.0
160.0
150.0
140.0
130.0
120.0
110.0
100.0
90.0
80.0
70.0
60.0
50.0
40.0
30.0
20.0
10.0
0
166.549
154.831
148.023
144.305
125.951
125.856
117.189
114.109
36.765
34.457
28.927
X : parts per Million : 13C
